# Reconstruction of Ancestral Protein Sequences Using Autoregressive Generative Models

**DOI:** 10.1093/molbev/msaf070

**Published:** 2025-03-26

**Authors:** Matteo De Leonardis, Andrea Pagnani, Pierre Barrat-Charlaix

**Affiliations:** DISAT, Politecnico di Torino, Corso Duca degli Abruzzi 24, Torino 10129, Italy; DISAT, Politecnico di Torino, Corso Duca degli Abruzzi 24, Torino 10129, Italy; Italian Institute for Genomic Medicine, IRCCS Candiolo, SP-142, Candiolo 10060, Italy; INFN, Sezione di Torino, Via Pietro Giuria 1, Torino 10125, Italy; DISAT, Politecnico di Torino, Corso Duca degli Abruzzi 24, Torino 10129, Italy

**Keywords:** co-evolution, ancestral sequence reconstruction, generative models

## Abstract

Ancestral sequence reconstruction (ASR) is an important tool to understand how protein structure and function changed over the course of evolution. It essentially relies on models of sequence evolution that can quantitatively describe changes in a sequence over time. Such models usually consider that sequence positions evolve independently from each other and neglect epistasis: the context-dependence of the effect of mutations. On the other hand, the last years have seen major developments in the field of generative protein models, which learn constraints associated with structure and function from large ensembles of evolutionarily related proteins. Here, we show that it is possible to extend a specific type of generative model to describe the evolution of sequences in time while taking epistasis into account. We apply the developed technique to the problem of ASR: given a protein family and its evolutionary tree, we try to infer the sequences of extinct ancestors. Using both simulations and data coming from experimental evolution we show that our method outperforms state-of-the-art ones. Moreover, it allows for sampling a greater diversity of potential ancestors, allowing for a less biased characterization of ancestral sequences.

## Introduction

Homologous proteins have a common evolutionary origin that can go back to billions of years. Throughout their evolution, they diversify through mutations while selection preserves their biological function. Consequently, many protein families contain thousands of sequences that are highly variable and yet maintain similar structures and functions. On the other hand, even a few mutations can destabilize a protein and destroy its function. A quantitative description how protein sequences change in time is thus a challenging problem, with important consequences for our understanding of the evolution of life.

Many probabilistic models of protein sequence evolution have been developed. Commonly used ones describe the evolution at each sequence position as a Markov chain across amino acid states, taking into account average properties of the substitution process such as more frequent transitions between similar amino acids (Dayhoff et al. 1978; Henikoff and Henikoff 1992; Jones et al. 1992). Variations in evolutionary speed at different sites are often represented by using a set of substitution rates to which sites can be assigned, usually coming from a Gamma distribution (Yang 1994). An important and widely accepted assumption is that sequence positions evolve independently. This has the advantage of greatly simplifying sequence evolution models, making them convenient to manipulate analytically and computationally manageable. However, it comes at the cost of ignoring epistasis, that is the fact that the effect of a mutation depends on the rest of the sequence.

Sequence evolution models are used in the general field of phylogenetics which explores the evolutionary relations between proteins. An notable application is that of ancestral sequence reconstruction (ASR): given a set of homologous sequences and their phylogenetic tree, ASR consists in inferring likely sequences for the internal nodes of the tree, which correspond to extinct ancestral proteins. Reconstructed proteins can then be synthesized and tested in the lab. The technique is used to study the sequence–function relationship in proteins, for instance by understanding which mutations cause a change in enzymatic activity or binding specificity of a protein (Wouters et al. 2003; Voordeckers et al. 2012; Hochberg and Thornton 2017). It can also be used to address fundamental evolutionary questions, such as the evolution reaction specificity or thermostability of proteins across the tree of life (Akanuma et al. 2013; Hobbs et al. 2012).

The large amount of protein sequence data combined with recent theoretical and computational work has also allowed the development of generative protein sequence models. These models build on the idea that the sequence variability among homologous protein with similar biological functions inform us about the sequence–function relationship. In practice, generative models are trained using large amounts of protein sequences and consist of a probability distribution P(s) over any potential amino acid sequence, with functional ones presumably being more probable. Classes of models include ones inspired from statistical physics such as the Potts model (Cocco et al. 2018) and restricted Boltzmann machines (Tubiana et al. 2019), or based on neural networks such as transformers (Rao et al. 2021; Ferruz et al. 2022). A major achievement of these models is the possibility of using them to sample new artificial sequences that are distant from any natural protein but still functional (Tian et al. 2018; Russ et al. 2020).

An essential ingredient for the success of generative models is the modeling of *epistasis*: the fact that the effect of a mutation on protein function depends on the rest of the sequence. Epistasis is caused by interaction between amino acids and is essential to describe the fitness landscape of a protein (Socolich et al. 2005; McGee et al. 2021). Interestingly, it has also been suggested that epistasis may be the cause of variable evolutionary rates across phylogenetic trees (de la Paz et al. 2020). Since common sequence evolution models ignore epistasis, they can only represent a crude approximation of the evolutionary constraints acting on a protein. As the change of a protein sequence in time depends on functional constraints, it is reasonable to expect that an inaccurate representation of the fitness landscape negatively affects the modeling of dynamics.

There has been effort in the phylogenetics community to develop models that take epistasis into account. For instance in Robinson et al. (2003) and Rodrigue et al. (2006), authors build an evolutionary model based on a structure-based fitness landscape. The evolutionary models obtained in this way can be used to detect the presence of epistasis and to show that including it leads to better fit of the data, but not to infer a phylogenetic tree or to reconstruct the states at internal nodes. Other approaches that perform phylogenetic inference under the assumption of coevolution make strong approximations such as the one of nonoverlapping pairs of coevolving sites (Meyer et al. 2019). Another promising direction is the use of generative models for phylogenetic tasks. However, the nonindependence of mutations that characterizes generative models makes it challenging to use them for dynamical purposes. Different studies have proposed using Potts models to describe evolutionary dynamics, but current techniques allow for little analytical treatment and are limited to forward simulation of sequences (Bisardi et al. 2021; Alvarez et al. 2024). Another promising approach is the use of variational autoencoders, which allow the representation of sequences in a continuous space (Moreta et al. 2021).

In this study, we set out to extend the application of generative models to describe evolutionary dynamics. First, we develop an analytically and numerically tractable sequence evolution model with generative properties, based on theso-called ArDCA generative model and its autoregressive architecture (Trinquier et al. 2021). Our model accounts for epistasis and is generative over long-term evolution but also allows use of some of the standard techniques used in phylogenetics such as e.g. Felsenstein’s pruning algorithm or an algorithm for irreversible models that we use here (Felsenstein 2003; Boussau and Gouy 2006). We then apply our model to ASR and demonstrate, using simulated data, that it outperforms state-of-the-art reconstruction techniques that assume independent sites, both when maximizing or sampling from the posterior. We use the program IQ-TREE (Minh et al. 2020) to compare to state of the art methods, and the list of methods that we use within IQ-TREE is detailed in the Methods section. Finally, we validate our approach with recent experimental data on directed evolution and show that reconstruction of a known ancestor is done more accurately than using a site-independent method. To our knowledge, this is the first use of such data to evaluate reconstruction methods.

## Results

### Autoregressive Model of Sequence Evolution

Models of evolution commonly used in phylogenetics rely on the assumptions that sequence positions evolve independently and that evolution at each position *i* follows a continuous time Markov chain (CTMC) parametrized by a substitution rate matrix $Q^{i}$. Matrix $Q^{i}$ is of dimensions q×q where q=4 for DNA, 20 for amino acids or 64 for codon models. The probability of observing a change from state *a* to state *b* during evolutionary time *t* is then given by $P_{i}(b\;|\;a,t)=(e^{tQ^{i}}{)}_{ab}$.

If the model is time-reversible, it is a general property of CTMCs that the substitution rate matrix can be written as


(1) $$Q=H\cdot \Pi =H\cdot \left(\begin{array}{ccc} \pi_{1} & 0 & 0\\ 0 & \ddots & 0\\ 0 & 0 & \pi_{q} \end{array}\right),$$

where H is symmetric with positive off-diagonal elements and Π is diagonal with positive entries that sum to 1 (Yang 2006). The diagonal elements of H are determined by requiring that the rows of Q sum to zero. The two matrices have simple interpretations. On the first hand, Π fixes the long-term equilibrium frequencies, that is $P_{i}(b\;|\;a,t)\underset{\left[t\to \infty \right]}{\to }\pi_{b}$. On the other, H influences the dynamics of the Markov chain but does not change the equilibrium distribution. Most commonly, both matrices are considered to be independent of the sequence position *i*, and H can potentially be scaled in order to represent different rates of evolutionary change (Yang 1994).

In order to incorporate constraints coming from a protein’s structure and function into the evolutionary model, we develop a protein family specific model of protein sequence evolution based on the autoregressive generative model ArDCA (Trinquier et al. 2021). Autoregressive models *à la* ArDCA build from the chain rule of conditional probabilities:


(2) $$\begin{array}{rl} P(a_{1},\ldots ,a_{L}) & =P(a_{1})P(a_{2}\;|\;a_{1})\ldots P(a_{L}\;|\;a_{1},\ldots ,a_{L-1})\\ & =\prod_{i=1}^{L}P(a_{i}\;|\;a_{<i}), \end{array}$$

where $a_{<i}=a_{1},\ldots ,a_{i-1}$ represents the amino acid states before position *i* and *L* is the length of the sequence. By construction, Equation (2) is an exact decomposition of the joint probability distribution of the sequence $a_{1},\ldots ,a_{L}$. There are L! such decompositions of *P*: for any permutation σ of the positions {1,…,L}, $P(a_{1},\ldots ,a_{L})=\prod_{i=1}^{L}P(a_{\sigma_{i}}\;|\;a_{<\sigma_{i}})$ is another exact decomposition of *P*.

ArDCA models the diversity of sequences in a protein family by proposing a specific functional form for conditional probabilities. In other words, the model is defined by *L* functions $p_{i}$ depending on parameters $\theta_{i}$ with the desired property


(3) $$p_{i}(a_{i}\;|\;a_{<i};\theta_{i})≃P(a_{i}\;|\;a_{<i}).$$

The precise functional form of $p_{i}(a_{i}\;|\;a_{<i};\theta_{i})$ is given in the Methods section. The model then assigns a probability $P^{AR}(a)$ to any sequence $a=\{a_{1},\ldots ,a_{L}\}$ of *L* amino acids:


(4) $$P^{AR}(a)=\prod_{i=1}^{L}p_{i}(a_{i}\;|\;a_{<i};\theta_{i}).$$

Note that since the model is trained on aligned sequences, states $a_{i}$ can include the gap symbol, which is treated as any other amino acid. Functions $p_{i}$ represent the probability according to the model to observe state $a_{i}$ in position *i*, given that the previous amino acids were $a_{1},\ldots ,a_{i-1}$. The set of parameters $\left\{\theta_{i}\right\}$ is learned by maximum likelihood using the aligned sequences of members of the family. Note that the autoregressive architecture is also employed in the context of deep-learning methods, to which the model we describe below could potentially be generalized (Ferruz et al. 2022; Madani et al. 2023). Deep autoregressive methods differ from ArDCA in that they use a more complex parametrization of $p_{i}$ and are usually trained on large set of unaligned proteins rather than a single family.

As explained above, the decomposition of Equation (2) is valid for any ordering of the sequence positions {1,…,L}. Each decomposition will lead to a different set of parameters $\left\{\theta_{i}\right\}$ and thus to a different generative model. The ordering used in ArDCA is not the natural {1,…,L} but rather an order where positions are sorted by increasing variability, which has been shown to give good generative capacities (Trinquier et al. 2021). For simplicity, we keep the notation of Equation (4): the position we call i=1 is not the first sequence position but rather the most conserved one, and so on until i=L which represents the most variable position.

It has been shown in Trinquier et al. (2021) that the generative capacities of ArDCA are comparable to that of state of the art models such as bmDCA (McGee et al. 2021). This means that a set of sequences sampled from the probability in Equation (4) is statistically hard to distinguish from the natural sequences used in training or, in other words, that the model can be used to sample new artificial homologs of a protein family. The generative capacities of a protein model comes from its ability to represent epistasis, that is the relation between the effect of a mutation and the sequence context in which it occurs. Here, epistasis is modeled through the conditional probabilities $p_{i}$: the distribution of amino acids at position *i* depends on the states at the previous positions {1,…i−1}.

We take advantage of the autoregressive architecture to define a generative evolutionary model. Given two amino acid sequences a and b, we propose that the probability of a evolving into b in time *t* take the form


(5) $$P(b\;|\;a,t)\overset{\text{def}}{\equiv }\;\prod_{i=1}^{L}q_{i}(b_{i}\;|\;a_{i},b_{<i},t),$$

where the position-specific conditional propagator $q_{i}$ is defined as


(6) $$\begin{array}{rl} q_{i}(b_{i}\;|\;a_{i},b_{<i},t) & ={\left(e^{t\cdot Q^{i}(b_{<i})}\right)}_{a_{i},b_{i}},\\ Q^{i}(b_{<i}) & =H\cdot \left(\begin{array}{ccc} \;p_{i}(1\;|\;b_{<i}) & 0 & 0\\ 0 & \ddots & 0\\ 0 & 0 & p_{i}(q\;|\;b_{<i}) \end{array}\right). \end{array}$$

According to these equations, evolution for each position *i* follows a standard CTMC. However, we use the decomposition of Equation (1) to set the equilibrium frequency at *i* to $p_{i}(b\;|\;b_{<i})$. In other words, we consider that position *i* evolves in the context of $b_{1},\ldots ,b_{i-1}$, and that its dynamics are constrained by its long-term frequency given by the autoregressive model. Compared to Equation (1), matrices H and Π now depend on the position *i* but also on the context $b_{<i}$. An important consequence of this choice is that our evolutionary model will converge at long times to the generative distribution $P^{AR}$:


(7) $$q_{i}(b_{i}\;|\;a_{i},b_{<i},t)\underset{\left[t\to \infty \right]}{\to }p_{i}(b_{i}\;|\;b_{<i}),\;P(b\;|\;a,t)\underset{\left[t\to \infty \right]}{\to }P^{AR}(b).$$

We argue here that such a property is essential to build a realistic protein sequence evolution model, particularly when considering evolution over long periods. Note that to converge to a generative distribution, accurate modeling of epistasis is required. Using site-specific frequencies would not be sufficient, as the effect of mutations in a protein sequence typically depends on the context (Socolich et al. 2005). The technique proposed here allows us to represent epistasis through the context-dependent probabilities $p_{i}$, while still considering each sequence position one at a time.

In the Methods section and in the Supplementary material online, we compute the transition rates associated to the propagator of Equation (5) and show that it can be seen as an approximation of dynamics in the fitness landscape defined by $P^{AR}$. It becomes exact at large times, as Equation (7) points out, and at small times. There are caveats to this approximation: our model has a nonreversible dynamic—although the context-dependent site propagators in Equation (6) are reversible—and in fact is not even a Markov process. Using non time-reversible evolutionary models is uncommon in the field, but this is mainly due to practical considerations and there are no fundamental reasons for evolution itself to be reversible (Felsenstein 2003). However, it is definitely out of the ordinary to model evolution with a non Markovian process. Another undesired consequence is that the generative distribution $P^{AR}$ is not stationary at all times in this process. This is in principle worrying, as it means that if dynamics are started from natural sequences, sequences generated at intermediate times could be nonfunctional according to the generative model.

These caveats are, to some extent, the price to pay to model epistasis on long time scales—see Equation (7)—while keeping an analytically tractable model. While definitely undesirable, they seem to have limited quantitative consequences: in supplementary fig. S1, Supplementary Material online, we show that deviations of the dynamics from the equilibrium $P^{AR}$ are quantitatively small. Another argument in this direction is the fact that reconstruction depends weakly on the placement of the root, indicating that the irreversibility of the model is not too strong (supplementary material Section B3, Supplementary Material online). Furthermore, the results that we present below show that our propagator improves ASR in different settings and can thus be seen as a useful approximation.

A final remark is that, as the ArDCA model itself, the proposed dynamic depends on the order in which decomposition Equation (2) is made. Indeed, a consequence of the autoregressive structure of the model is that the first position treated by the model (i=1) “evolves” independently from the context, while the last one depends on all the rest of the sequence. In practice, it is difficult to say whether a given ordering better describes biological evolution: there is an astronomically large number of permutations L!, and there is no obvious direct measure of whether one better fits evolutionary dynamics. For this reason, we make the simplifying choice of only considering the ordering by increasing diversity of sites, which has been found in (Trinquier et al. 2021) to have good generative capacities.

We underline that this approach has important differences with standard models of evolution used in phylogenetics. In phylogenetic reconstruction, the tree and the sequence evolution model are usually inferred at the same time and from the same data. The number of parameters of the evolutionary model is then kept low to reduce the risk of overfitting, for instance by using a predetermined set of evolutionary rates to account for variable and conserved sites. Methods that introduce more complex models such as site-specific frequencies do so by jointly inferring the parameters and the tree, leading to computationally intensive algorithms (Halpern and Bruno 1998; Puller et al. 2020).

Here instead, parameters of the generative model in Equation (4) are learned from a protein family, i.e. a set of diverged homologous protein sequences. While it is true that these sequences share a common evolutionary history and cannot be considered as independent samples, common learning procedures only account for this in a very crude way (Cocco et al. 2018; Trinquier et al. 2021). Despite this, it appears that the generative properties of such models are not strongly affected by the phylogeny (Hockenberry and Wilke 2019; Rodriguez Horta and Weigt 2021). This allows us to proceed in two steps: first construct the model from data while ignoring phylogeny, and then use it for phylogenetic inference tasks.

An advantage of this approach is that once the model of Equation (4) is inferred, the propagator in Equation (5) comes “for free” as no additional parameters are required. Importantly, our model does not use site-specific substitution rates. Indeed, it has been shown that these can be seen as emergent properties of more complex models of evolution (de la Paz et al. 2020). However, a constraint is that the inference of the generative model requires the existence of an appropriate training set, that is a protein family with sufficient variability among its members.

### Ancestral Sequence Reconstruction

We apply our evolutionary model to the task of ASR. The goal of ASR is the following: given a set of extant sequences with a shared evolutionary history and the corresponding phylogenetic tree, is it possible to reconstruct the sequences of extinct ancestors at the internal nodes of the tree? Along with the autoregressive evolutionary model described above, we thus need two inputs to perform ASR: a known phylogenetic tree, and the multiple sequence alignment of the leaf sequences. The length of the aligned sequences has to exactly correspond to that of the autoregressive model.

The reconstruction with the autoregressive model proceeds as follows.

- For position i=1, we use the evolutionary model defined by the equilibrium frequencies $p_{1}$ to reconstruct a state $a_{1}^{n}$ at each internal node *n* of the tree. For i=1, the transition rate matrix $Q^{1}$ as defined in Equation (6) depends only on $p_{1}$, which in turn does not depend on the context. For a branch of length *t*, the transition probabilities between two states *a* and *b* is $q_{1}(b\;|\;a,t)=(e^{tQ^{1}}{)}_{ab}$.
- Iterating through subsequent positions i>1: we reconstruct state $a_{i}^{n}$ at each internal node *n* using the model defined in Equation (6), with the context $a_{<i}^{n}$ having been already reconstructed in the previous iterations. The procedure is the same as the i=1 case, the only difference being that the transition rate matrix $Q^{i}$ now also depends on the context at positions 1,…,i−1.

It is important to note that when any position i>1 is reconstructed, the context at different internal nodes of the tree may differ. For a branch joining two nodes (n,m) of the tree, the evolutionary model will thus differ if we go down or up the branch: in one case the context at node *n* must be used, in the other case the context at node *m*. This is the cause of the time-irreversibility of the model. For this reason, we compute the probability of reconstructions using an algorithm adapted to irreversible models (Boussau and Gouy 2006), described in details in supplementary material Section A, Supplementary Material online.

Using this technique we obtain, for any internal node *n* and any alignment position *i*, the posterior probability $P(a_{i}^{n}\;|\;T,D)$ of the amino acid state $a_{i}^{n}$ given the tree T and the sequences at the leaves D. This probability is computed by marginalizing over other the states of other internal nodes. We call *maximum a posteriori* reconstruction (MAP) the state obtained by maximizing $P(a_{i}^{n}\;|\;T,D)$. In this case, each iteration reconstructs the most probable residue at position *i* for all internal nodes of the tree. Alternatively, states of internal nodes can be sampled from $P(a_{i}^{n}\;|\;T,D)$ to obtain a *posterior sampling* reconstruction. In any case, our reconstruction is marginal: the posterior at a node is obtained by marginalizing over the states of other nodes. While it is in principle possible to extend it to joint reconstruction, as explained in (Boussau and Gouy 2006), we have not implemented it and do not consider it in this work.

In any realistic application, the phylogenetic tree has to be reconstructed from the aligned sequences. In principle, a consistent approach would use the same evolutionary model for tree inference and ASR. However, our model does not allow us to reconstruct the tree. Therefore, in any realistic application, the tree is reconstructed using an evolutionary model that typically will differ from ours. To reduce issues related to this evolutionary model discrepancy, we adopt the following strategy: our ASR method blindly trusts the topology of the input tree, but recomputes the branch length using the sequences. As explained in the Methods, there is no direct way to optimize branch length with the autoregressive model. For simplicity, we use a profile model with position-specific amino acid frequencies for this task. This provides a relatively accurate estimate of the branch lengths, as shown in supplementary fig. S4, Supplementary Material online.

A consequence of the irreversibility of the evolutionary model is that the reconstruction potentially depends on the placement of the root of the tree. This is not an issue in the results that follow since we work with simulated trees for which the root is known exactly. However, it may be a concern when applying this to biological datasets. In supplementary material Section B3, Supplementary Material online, we explore the effect of root placement on the reconstruction. Results are overall reassuring, with the difference between reconstructions remaining below a Hamming distance of 0.5% even for large errors in root placement.

### Results on Simulated Data

There are two difficulties when evaluating the capacity of a model to perform ASR. The first is that in the case of biological data, the real phylogeny and ancestral sequences are usually not known. As a consequence, one must rely on simulated data to measure the quality of reconstruction. The second is that the reconstruction of an ancestral sequence is always uncertain, as evolutionary models are typically stochastic. The uncertainty becomes higher for nodes that are remote from the leaves. This means that it is only possible to make a statistical assessment about the quality of a reconstruction.

To test our approach, we adopt the following setup. We first generate phylogenetic trees by sampling from a coalescent process. We decide to use Yule’s coalescent instead of the more common Kingman. The latter tends to produce a large majority of internal nodes in close vicinity to the leaves with the others separated by very long branches, resulting in a trivial reconstruction for most nodes and a very hard one for the deep nodes. Yule’s coalescent generates a more even distribution of internal nodes depths (defined as the distance to the closest leaf), allowing us to better evaluate reconstruction quality, see Supplementary material online and supplementary fig. S5, Supplementary Material online. For each tree, we simulate the evolution of sequences using a model that we refer to as “evolver” to obtain two multiple sequence alignments, one for the leaves and one for the internal nodes of the tree. We then reconstruct internal nodes using the desired approach by using the leaf alignment and the tree topology as input data.

We will consider two kinds of evolver models: (i) the same autoregressive model that we will then use for reconstruction, which is an ideal case and (ii) an evolutionary model based on a Metropolis sampling of a Potts model. These two evolvers come from models trained on actual protein families: we use evolvers based on the PF00072 response regulator family for results of the main text, and show results for three other families (PF00014, PF00076, and PF00595) in the Supplementary material online (see Table 1 for details on these three other families). It is important to note that the approach that we propose only makes sense when considering the evolution a protein family on which the model in Equation (4) is trained. Hence, any evolver model used in our simulations should reproduce at long times the statistics of the considered protein family, i.e. it should satisfy Equation (7). For this reason, we only consider the two evolvers above and do not use more traditional evolutionary models such as an arbitrary GTR on amino-acids (Rambaut and Grass 1997).

**Table 1.** Protein families used in this work.

			IQ-TREE model	
Family		Alignment length	ArDCA	Potts
PF00014	Trypsin inhibitor Kunitz domain	53	PMB+R3	PMB+I+G4
PF00072	Response regulator receiver domain	112	PMB+I+G4	PMB+I+G4
PF00076	RNA recognition motif	70	PMB+R3	PMB+I+G4
PF00595	PDZ domain	82	PMB+R3	PMB+R5
PF13354	Beta-lactamase enzyme	214	JTT+G4	

The last two columns give the best hit models found by IQ-TREE, for the two different evolvers (autoregressive and Potts).

For reconstruction, we compare our autoregressive approach to the commonly used IQ-TREE program (Minh et al. 2020) with the flag -m MFP to use the ModelFinder (Kalyaanamoorthy et al. 2017). In this mode, when supplied with a protein sequence alignment and a tree, IQ-TREE infers a joint substitution rate matrix for all sequence positions. Because the best evolutionary model found may differ when using two different alignments, we pick for each family the model most commonly found by IQ-TREE across a reduced range of simulations (Methods). The list of models found and used in our analysis is reported in Methods (Reconstruction with IQ-TREE section): in most cases, the PMB matrix was used (Veerassamy et al. 2003), with different options for across-sites rate variability (+I+G4 or +I+R3). Ancestral states are then reconstructed using an empirical Bayesian method (Yang et al. 1995). We either selected the state corresponding to the maximum of the posterior (MAP) or sampled from the posterior. In the extra analysis of the Supplementary material online, we also use the flag +C60 to perform reconstruction using profile mixture models (Si Quang et al. 2008). As for the autoregressive model, we provide the topology of the real tree to IQ-TREE and let it recompute the branch lengths.

####  

##### Autoregressive evolver

**Fig. 1.**
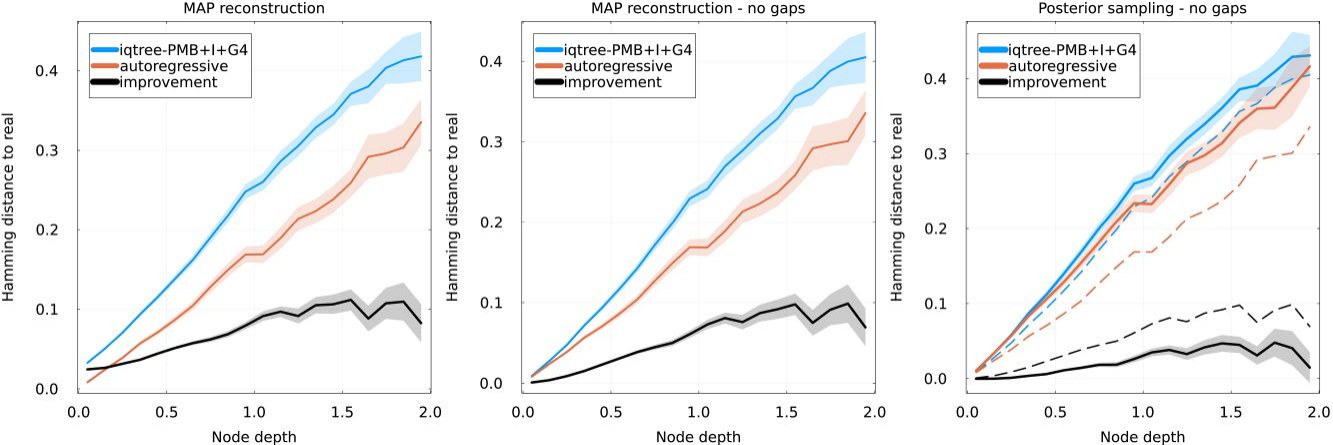
Hamming distance (normalized by sequence length) between reconstructed and real sequences as a function of node depth, defined as the distance from the node to the closest leaf in the “ground-truth” tree used to simulate the data. Reconstruction if performed using IQ-TREE and our autoregressive approach, with the evolutionary model used by IQ-TREE reported in the legend. The difference between the two methods (“improvement”) is shown as a black curve. Estimation of the uncertainty is shown as a ribbon. The evolver and reconstruction autoregressive models are learned on the PF00072 family. Left: Hamming distance between the full aligned sequences, gaps included, using maximum a posteriori reconstruction. Center: Hamming distance ignoring gapped positions, using MAP reconstruction. Right: comparison of posterior sampling (solid lines) and MAP (dashed lines) reconstructions, ignoring gaps.

We first investigate the case of the autoregressive evolver. This setting is of course ideal for our method, as there is perfect coincidence between the model used to generate the data and to perform ASR. We first evaluate the quality of reconstruction by computing the Hamming distance of the real and inferred sequences for each internal node of the simulated phylogenies. The left and central panels of Fig. 1 (supplementary fig. S10, Supplementary Material online for additional families) show this Hamming distance as a function of the node depth, that is the distance separating the node from the leaves along the branches of the tree on which evolution was simulated, and for a MAP reconstruction. Hamming distance is computed including gap characters in the aligned sequences on the right panel, while they are ignored on the central one, and is normalized by the length of the sequence: a distance of 1 would thus indicate entirely different sequences. We see that the autoregressive reconstruction clearly outperforms the state of the art method: the improvement in Hamming distance increases with node depths, and the distance to the real ancestor drops from ∼0.4 to ∼0.3 when using the autoregressive approach. The increase in reconstruction quality with node depths is consistent with recent findings that epistasis only becomes important at relatively large sequence divergences (Park et al. 2022; Di Bari et al. 2024).

Interestingly, the performance of IQ-TREE degrades if Hamming distance is computed including gaps, as in the left panel. This is because like other popular methods, IQ-TREE treats gaps in input sequences as unknown amino acids, and reconstructs an ancestral amino acid for gapped positions (Yang 2007; Minh et al. 2020). On the contrary, our autoregressive approach, like many generative models, treats gaps as if they were an additional amino acid and will reconstruct ancestral sequences that can contain gaps. This effect is particularly visible at low node depths and benefits the autoregressive approach as aligned ancestral sequences can in fact contain gaps. Considering gaps as an additional amino acid is an advantage in our setup, as both evolvers use this convention. However, it is not clear that this advantage extends to real biological data, as the insertion–deletion processes during evolution may not be accurately captured by our model. For this reason, we also show the performance of reconstruction when ignoring the effects of gaps in the Hamming distance. This also leads to a smaller but clear improvement when using the autoregressive approach as shown in the central panel.

The right panel of Fig. 1 shows the quality of the reconstruction when reconstructing by sampling the posterior. In this case, an ensemble of sequences is reconstructed for each internal node, and the metric is the average Hamming distance between this ensemble and the real ancestor. Gaps are again ignored when computing the Hamming distance. We again observe an improvement when using the autoregressive method, of slightly lesser magnitude than in the MAP case.

To understand how these results depend on the complexity of the evolutionary model used by IQ-TREE, we extend the comparison to reconstruction using the profile mixture models proposed by IQ-TREE (Si Quang et al. 2008). In our case, we use the C60 flag to have IQ-TREE infer 60 different site-specific profiles, with the likelihood at each site being averaged over these profiles. Results are shown in supplementary fig. S7, Supplementary Material online (supplementary fig. S11, Supplementary Material online for additional families). It is clear that the profile model improves IQ-TREE’s reconstruction, as the improvement now peaks at a Hamming distance of approximately 0.06 instead of 0.1 in Fig. 1. However, the performance of the autoregressive reconstruction remains consistently above the independent model.

##### Properties of reconstructed sequences

**Fig. 2.**
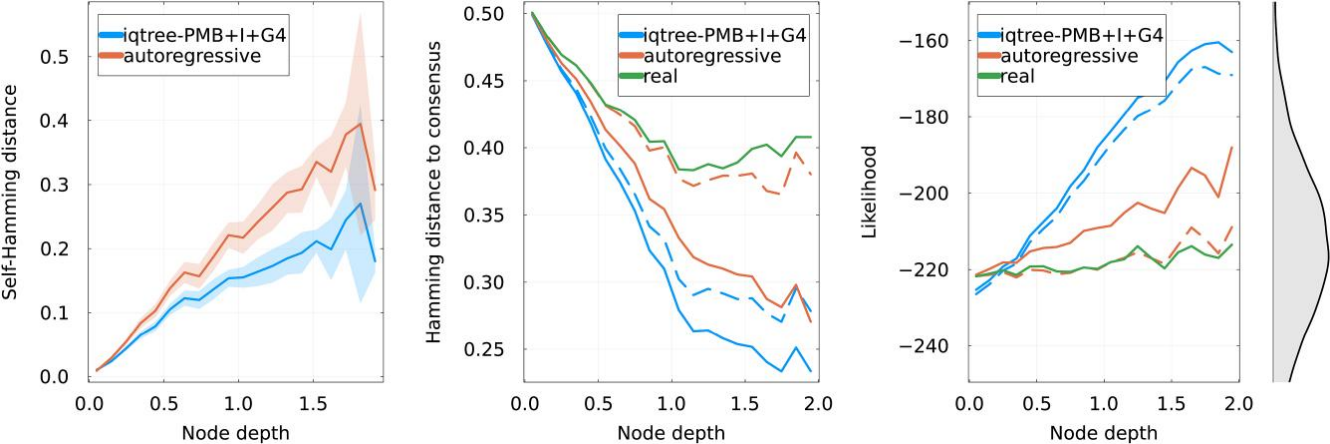
Left: For posterior sampling reconstruction, average pairwise normalized Hamming distance among sequences reconstructed for each internal node. This quantifies the diversity of possible ancestral reconstructions. Center: Normalized Hamming distance between reconstructed sequences and the consensus sequence of the alignment. Solid lines represent MAP reconstruction or the real internal sequences, and dashed lines posterior sampling. IQ-TREE appears more biased towards the consensus sequence. Right: Log-likelihood of reconstructed and real sequences in the autoregressive model, i.e. using the logarithm of Equation (4). MAP methods (orange and blue solid lines) are biased towards more probable sequences. Posterior sampling autoregressive reconstruction gives sequences that are at the same likelihood level than the real ancestors. The equilibrium distribution of likelihood of sequences generated by Equation (4) is shown on the right.

To further analyze the reconstructed sequences, we first look at the diversity of generated ancestors when sampling the posterior. The left panel of Fig. 2 (supplementary fig. S12, Supplementary Material online for additional families) shows the average normalized Hamming distance between sequences reconstructed at the same internal node, as a function of depth. For deeper nodes (depth ≳1), the autoregressive approach reconstructs a significantly more diverse set of sequences than IQ-TREE: Hamming distance between reconstructions saturates at 0.2 for the latter, while it steadily increases for the former. Higher diversity can be interpreted as a greater uncertainty concerning the ancestral sequence. However, this must be put in the context of Fig. 1: sequences obtained by autoregressive reconstruction are more varied but also on average closer to the real ancestor.

The difference in sequence diversity for the two methods is in part explained by the central panel of Fig. 2, which shows the Hamming distance between reconstructed ancestors and the consensus sequence of the multiple sequence alignment at the leaves. It appears there that for deep nodes, IQ-TREE reconstructs sequences that are relatively similar to the consensus, with an average distance between the posterior sampling reconstruction and the consensus of about 0.3. Contrasting with that, results of the autoregressive method shows less bias towards the consensus with an average distance of 0.4 for deep nodes, in line with the real ancestors. We also note that MAP sequences for both method are always closer to the consensus than sampled ones, a bias that had already been observed (Williams et al. 2006).

The bias induced by ignoring the equilibrium distribution of the sequences is also visible in the right panel of Fig. 2: it shows the log-likelihood of reconstructed and real ancestral sequences according to the generative model. Note that the log-likelihood here comes from the log-probability of Equation (4) and can be interpreted as the “quality” of a sequence according to the generative model. It is unrelated to the likelihood computed in the phylogenetic reconstruction algorithm. Reconstructions with IQ-TREE increase in likelihood when going deeper in the tree, eventually resulting in “too good” sequences that are very uncharacteristic of the equilibrium generative distribution as can be seen from the histogram on the right. This effect also happens with the MAP reconstruction of the autoregressive model, although to a lesser extent. The autoregressive reconstruction obtained from sampling the posterior does not suffer from this bias and reconstructs sequences with a log-likelihood that is similar to that of the real ancestors. Interestingly, IQ-TREE’s reconstruction using a profile model suffers less from these biases, as can be seen in supplementary figs. S8 and S13, Supplementary Material online. This suggests that having a more precise evolutionary model tends to reduce biases in the reconstruction.

##### Potts evolver

We assess the performance of our reconstruction method in the case where the evolver is a Potts model. Potts models are a simple type of generative model and have been used extensively to model protein sequences. They can be used to predict contact in three dimensional structures, effects of mutations, protein–protein interaction partners (Cocco et al. 2018). They can be sampled to generate novel sequences which are statistically similar to natural ones and often functional (Russ et al. 2020; Alvarez et al. 2024). Additionally, it has recently been shown that they can be used to describe the evolution of protein sequences both qualitatively and quantitatively (Bisardi et al. 2021).

Potts and autoregressive models both accurately reproduce the statistical properties of protein families. In this sense, they correspond to similar long-term generative distributions in the sense of Equation (7). However, the dynamics of a Potts model are fundamentally different from the ones of usual evolutionary models, including our autoregressive one. Indeed, they are described by a *discrete* time Markov chain, instead of the continuous time used in models based on substitution rate matrices such as in Equation (1) (Alvarez et al. 2024). For Metropolis steps which we use here, the discrete time corresponds to attempts at mutation which can be either accepted or rejected depending on the effect of the mutation according to the model. These dynamics naturally give rise to different evolutionary timescales for various sequence positions, as well as interesting qualitative behavior such as the entrenchment of mutations (Di Bari et al. 2024).

**Fig. 3.**
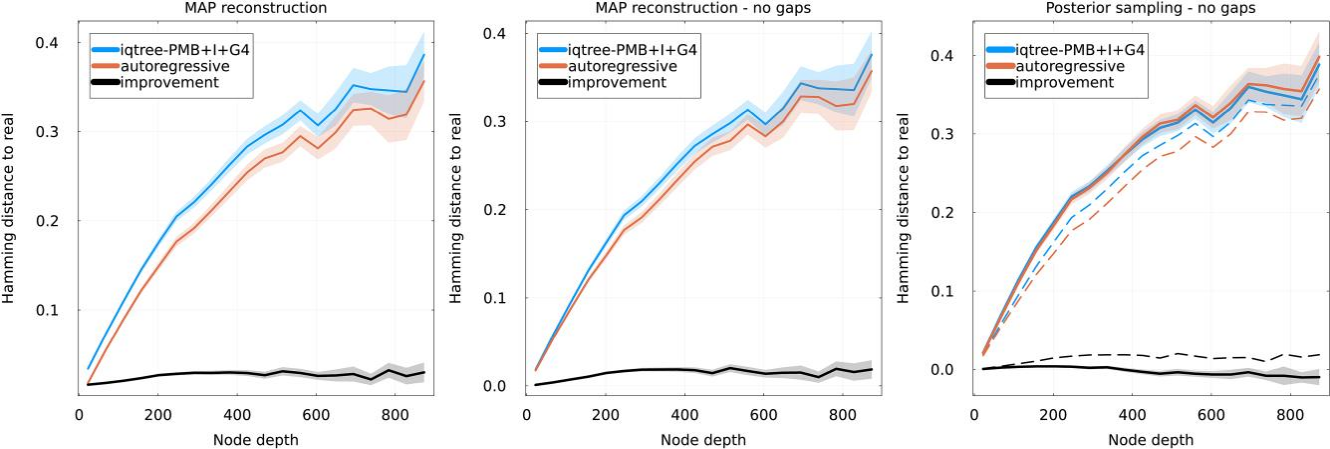
Analogous to Fig. 1, but using a Potts model as the evolver. Normalized Hamming distance between reconstructed and real sequences as a function of node depth, using IQ-TREE and our autoregressive approach. The difference between the two methods is shown as a black curve. The evolver and reconstruction autoregressive models are learned on the PF00072 family. Left: Normalized Hamming distance between the full aligned sequences, gaps included, using MAP reconstruction. Center: Normalized Hamming distance ignoring gapped positions, using MAP reconstruction. Right: comparison of posterior sampling (solid lines) and MAP (dashed lines) reconstructions, ignoring gaps.

To see how this change in dynamics affects our results, we (i) sample a large and varied ensemble of sequences from the Potts model and use it to train an autoregressive model, in a way to guarantee consistent long-term distributions between the Potts and autoregressive, and (ii) evolve the Potts model along random phylogenies, generating alignments for the leaves and the internal nodes in the same way as above. We then attempt reconstruction of internal nodes using the inferred autoregressive model and IQ-TREE. Figure 3 shows the results of reconstruction, with panels directly comparable to Fig. 1. We again see a consistent improvement when using the autoregressive model over IQ-TREE, although of a much smaller amplitude, with an absolute improvement gain in Hamming distance of about 2% for deep internal nodes.

### Results on Experimental Evolution Data.

We take advantage of recent developments in directed evolution experiments to test our method in a controlled setting. We use the data published in Stiffler et al. (2020): in this work, authors evolved the antibiotic resistant proteins *β*-lactamase PSE-1 and acetyltransferase AAC6 by submitting them to cycles of mutagenesis and selection for function. Starting from a wild-type protein, they obtained thousands of diverse functional sequences after the directed evolution. An interesting result of this work is that it is possible to recover structural information about the wild-type from the set of evolved sequences.

Here, we use these data as a test setting for ASR: the sequences obtained after directed evolution all derive from a common ancestor, the wild-type, of which we know the amino acid sequence. We can thus reconstruct the wild-type sequence using different ASR methods and compare it to the ground truth. The phylogeny is not known, but given the large population size during the experiment and the relatively low number of selection rounds, it is reasonable to approximate it using a star-tree, i.e. a tree with a single coalescent event taking place at the root (see Methods). Since the reconstruction task is most interesting when using relatively varied sequences, we decide to use data for the PSE-1 wild-type where 20 cycles of mutagenesis and selection have been performed, resulting in a mean Hamming distance of 12% to the wild-type.

Our ASR procedure is as follows. We randomly pick the amino acid sequences of *M* proteins among the ones evolved from PSE-1 after 20 cycles of mutagenesis and selection, with 3≤M≤640. The total number of sequences at round 20 of directed evolution is much larger, making it computationally hard to use all of them. We then construct a star-like phylogeny and place the *M* selected sequences at the leaves and perform ASR using either IQ-TREE or our autoregressive method which we have trained on an alignment of PSE-1 homologs. We obtain the reconstructed amino acid sequence of the root, which we can then compare to the actual wild-type. As a comparison, and because our approximation of the phylogeny is very simple, we also attempt to reconstruct the root by taking the consensus sequence of the *M* leaves. We repeat this procedure 100 times for each value of *M* for a statistical assessment of the different methods.

**Fig. 4.**
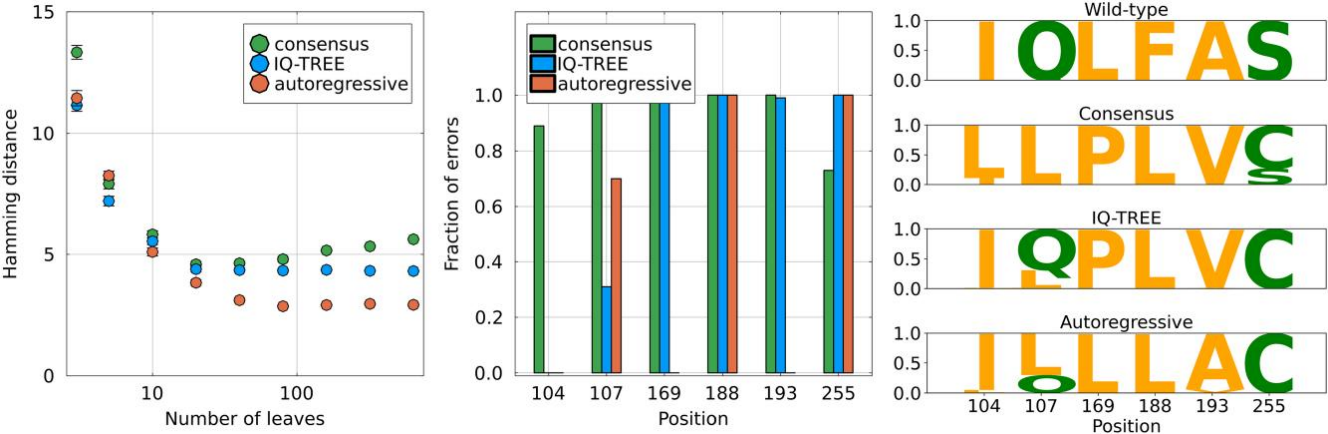
Reconstruction of the wild-type PSE1 sequence used in Stiffler et al. (2020) using sequences from round 20 of the directed evolution. Left. Nonnormalized Hamming distance to the wild-type PSE1 sequence as a function of the number of sequences *M* used for reconstruction. The fact that the consensus method has a local minimum is discussed in the Supplementary material online. For comparison, the average distance between a leaf sequence and the wild-type is 25. The error bars are computed using the standard deviation obtained from the 100 choices of sequences. Middle. For the six sequence positions where most of the reconstruction errors are located, fraction of errors of each method out of 100 independent reconstructions using different sets of M=640 leaves. Right. Sequence logo of the reconstructed sequence for the three methods, obtained using 100 independent reconstructions with different sets of M=640 leaves. The logo is only shown for the six positions where most errors are located. For example, all three methods fail 100 times at position 147, reconstructing a leucine *L* instead of a phenylalanine *F*.

The results are shown in Fig. 4. The left panel shows the average nonnormalized Hamming distance to the wild-type as a function of the number of leaves used *M*. For a low *M*, all methods understandably make a large number of errors, with a mean Hamming distance larger than 10 for M=3. For a higher *M*, IQ-TREE and the autoregressive method stabilize to a fixed number of errors: we find a Hamming distance of ∼4.3 for IQ-TREE and ∼2.9 for the autoregressive. The consensus curiously reaches a minimum at intermediate *M*, a fact discussed in the Supplementary material online, and saturates at a Hamming distance of 6 when considering all sequences of the round 20. The reconstruction errors are overwhelmingly located at six sequence positions. In the central panel, the fraction of mistakes made at these six positions over the 100 repetitions of M=640 leaves is shown for each method. We observe that there are two positions (169 and 193) where IQ-TREE systematically fails at recovering the wild-type state while the autoregressive model’s reconstruction is correct. Interestingly, the corresponding mutations are considered beneficial by the ArDCA model, see supplementary fig. S6, Supplementary Material online. Inversely, IQ-TREE recovers the wild-type state more often at position 107. The right panel shows the logo of the set of reconstructed sequences at these 6 positions and for each method.

Overall, we see that the reconstruction of the autoregressive model is more accurate. This gain in accuracy comes from the representation of the functional constraints acting on the PSE-1 protein by the generative model, which are inferred separately using an alignment of homologs. The improvement in reconstruction errors is modest, going from an average Hamming distance of 4.3 to 2.9. However, the gain is intrinsically limited by the data itself: the evolved sequences have an average Hamming distance of about 12% to the ancestor, which is experimentally challenging but remains small compared to the divergence found in the homologs of PSE-1. For instance, the root-to-tip distance estimated by IQ-TREE and the autoregressive model are respectively 0.13 and 0.15, corresponding to the regime of shallow trees when comparing with Fig. 1.

## Discussion

The reconstruction of ancestral protein sequences has long been a cornerstone of evolutionary biology, helping to elucidate the mechanisms of protein function and evolution over billions of years. The accuracy of ASR has profound implications not only for our understanding of evolution but also for practical applications in synthetic biology and proteins engineering. However, the widely used models in phylogenetics often rely on the assumption of independent sequence evolution at different positions, neglecting epistatic interactions that play a crucial role in determining protein function. This simplification limits their ability to accurately capture the full complexity of evolutionary dynamics.

In this study, we addressed this limitation by developing a novel generative model based on the ArDCA autoregressive framework, which explicitly accounts for epistasis, an essential factor in protein evolution. By incorporating the dependencies between amino acids within sequences, our model offers a more realistic description of protein evolution, capturing the nonindependence of mutations over time. A significant contribution of this work is extending the application of generative models to cope with phylogenetic constraints. Our model not only preserves the generative capacity over long-term evolution but it also enables the use of classical phylogenetic techniques normally restricted to independent-site models. The ability to integrate generative context-aware models into these established algorithms represents a substantial advance, allowing for more accurate inference of evolutionary relationships and ancestral states. This, besides the theoretical interest in ASR, is a powerful tool to help us understanding how phylogenetic constraints impact the structure and/or the function of the protein of interest.

Our evaluation of the model using simulated data demonstrated that it outperforms IQ-TREE, a state-of-the-art tool for ASR, in reconstructing ancestral sequences. This improvement highlights the importance of incorporating epistasis into evolutionary models, as ignoring these interactions likely leads to less accurate reconstructions. Furthermore, we validated our approach using experimental data from directed evolution experiments. These data offer a unique opportunity to test the accuracy of ASR methods, and our model achieved more accurate reconstructions of known ancestors compared to IQ-TREE, underscoring the robustness of our approach.

Using the generative nature of our model, we can sample sequences at internal nodes that should in principle remain functional despite being distant from any naturally occurring protein. Most ASR studies have used maximum a posteriori or maximum likelihood reconstructions, as Bayesian reconstructions are more often found to accumulate deleterious mutations and can be nonfunctional (Hobbs et al. 2012; Eick et al. 2017). At the same time, the most likely solution can be biased and may be unrepresentative of the phenotype of the real ancestor, leading to incorrect biological conclusions (Williams et al. 2006; Wheeler et al. 2016). We ourselves observe these biases in our simulations, in the form of a convergence to the consensus sequence and an unnaturally high likelihood according to the generative model. Being able to propose an ensemble of sequences sampled from a generative model at each internal node could thus lead to more robust biological conclusions about ancestral life.

Another feature of our model is the way it models gaps. IQ-TREE, as well as many other phylogenetic reconstruction methods, treats alignment gaps as missing information, and will reconstruct amino-acid states at these positions (Yang 2007; Minh et al. 2020). In contrast, most alignment based generative models such as ArDCA treat gaps as a particular state that a position can be in, on equal footing with other amino acids (McGee et al. 2021; Trinquier et al. 2021; Rao et al. 2021). This can have drawbacks when modeling evolution, as the dynamics of insertions–deletions and of point mutations can be quite different (Feinauer et al. 2014). However, being able to model gaps during ancestral reconstruction likely increases accuracy, as there is no reason to think that ancestral sequences would align to extant ones without any gaps.

Despite its good performance, our model comes with several caveats. First, our ad hoc way to infer branch lengths is not ideal and differs from standards used in the field. The method would clearly benefit from improvements in this direction. More importantly, the nature of our approximation has unsatisfying consequences, as the dynamic is non Markovian, irreversible, and does not remain at equilibrium with the generative model at all times. As evolution in an epistatic landscape is particularly challenging to model and requires some kind of approximation. We think our method should be considered as such: a useful approximation that allows incorporating context-dependence in phylogenetic models while remaining analytically and numerically tractable. The quantitative consequences of its undesirable properties are limited, as shown in the supplementary analysis on root placement and on the out-of-equilibrium dynamics. Overall, our results show that the benefits of the method outweigh its disadvantages.

The success of our model in both simulations and experimental validation suggests that generative models with autoregressive architectures are powerful tools for studying the dynamics of protein sequence evolution. By capturing the intricacies of epistatic interactions, our model not only improves the accuracy of ancestral sequence reconstruction but also provides new insights into the underlying evolutionary processes. Future work could explore the application of this model to other protein families and further refine the methodology to enhance its applicability in broader phylogenetic contexts.

In conclusion, the integration of epistasis into evolutionary models represents a necessary and timely advancement for the field. Our generative model provides a more nuanced understanding of protein evolution, paving the way for more accurate reconstructions of ancestral sequences and a deeper exploration of the evolutionary dynamics that shape the diversity of life.

## Methods

### ArDCA

The ArDCA model assigns a probability to any sequence of amino acids of length *L* given by


(8) $$P^{AR}(a)=\prod_{i\in \sigma (L)}p_{i}(a_{i}\;|\;a_{<i}),$$

where σ(L) is a permutation of the *L* first integers and $a_{<i}$ stands for $a_{1},\ldots ,a_{i-1}$. This means that the order in which the conditional probabilities $p_{i}$ are applied is not necessarily the sequence order. The permutation *σ* is fixed at model inference.

Following (Trinquier et al. 2021), we model the conditional probabilities $p_{i}$ as:


(9) $$p_{i}(b\;|\;a_{<i})=\frac{1}{Z_{i}}\exp\;\left(\sum_{\;j<i}J_{ij}(b,a_{j})+h_{i}(b)\right),$$

with the *i q*-dimensional vectors $J_{i\cdot }$ and $h_{i}$ are learned parameters. It is worth observing that the proposed parametrization of the conditional probabilities $p_{i}$ enables an efficient parameters learning by likelihood maximization. In the machine learning community, this particular parametrization is known as the soft-max regression (Hastie 2009), which is the generalization to multiclass class regression of the standard logistic regression. The model is normally trained using a multiple sequence alignment of homologous proteins, i.e. a protein family, by finding the parameters *J* and *h* that maximize the likelihood of the sequences. It was shown in Trinquier et al. (2021) that this specific parametrization captures essential features of the variability of members of a protein family.

By definition, homologous proteins share a joint evolutionary history and cannot be considered as statistically independent. To avoid biases, a reweighting is applied to sequences based on their vicinity to other sequences. This scheme has been showed to substantially increase the performance of such models (Cocco et al. 2018).

### Approximative Nature of the Propagator

The autoregressive propagator defined in Equation (5) is practical because it allows computation of the transition probability between any two sequences and for any time. However, it is only an approximation of the dynamics, as we will show below. The full derivation of these results can be found in supplementary material Section B2, Supplementary Material online.

The propagator that we would ideally like to use would (i) be Markovian and time reversible and (ii) have the generative model $P^{AR}$ as its stationary distribution. It is possible to derive a transition rate matrix Q that has these properties (Supplementary material online):


(10) $$Q_{ab}=\mu \left\{ \begin{array}{c} 0\;\text{if}\;a\;\text{and}\;b\;\text{differ at more than two sites,}\\ p_{i}(b_{i}\;|\;a_{<i})\;\text{if}\;a\;\text{and}\;b\;\text{differ only at site}\;i,\;\\ \sum_{i=1}^{L}(p_{i}(a_{i}\;|\;a_{<i})-1)\;\text{if}\;a=b,\; \end{array}\right.$$

where a and b are any two sequences and *μ* is a scalar rate. Note that the transition rate here is from sequence to sequence, and Q is of dimensions $q^{L}\times q^{L}$ with q=21 the number of amino-acid states plus the gap symbol. The corresponding transition probability matrix $P^{\prime }$ would be defined by


(11) $$P^{\prime }(b\;|\;a,t)=(e^{tQ}{)}_{ab}.$$

The main issue is that because of the dimensions of Q, and because we are incapable of calculating its eigenvectors and eigenvalues, $P^{\prime }$ cannot be used in practice. There exist workarounds if the goal is to sample from $P^{\prime }$ (Robinson et al. 2003; Nasrallah et al. 2011). However, they are not applicable to the task of ASR.

Our autoregressive propagator *P* has two properties that make it an attractive approximation. First,


(12) $$P(b\;|\;a,t)\underset{\left[t\to \infty \right]}{\to }P^{AR}(b),$$

meaning that it has the right stationary distribution at long times. Informally, we can write $P≃P^{\prime }$ for t→∞. Secondly, in the case where matrix H of Equation (1) has uniform off-diagonal terms equal to *μ*, the derivative of *P* with respect to time at t=0 happens to be the Q of Equation (10). Therefore,


(13) $$P(b\;|\;a,t)\underset{t\to 0}{∼}\;{\left(1+tQ\right)}_{ba},$$

where 1 is the identity. This means that for small times, *P* and $P^{\prime }$ are equal up to order one in *t*. Our *P* is therefore an approximation of the desired $P^{\prime }$, which becomes exact at small and large times.

Even though we have shown in the text that it gives good results, there are caveats to this approximation. The first is that our propagator does not define a Markovian dynamic and is also time irreversible. The second is that it does not remain in equilibrium with the generative $P^{AR}$ at intermediate times. However, the approximation can still be useful if deviations from equilibrium are not too large. In the Supplementary material online, we show that sequences generated from P(b|a,t) when starting from an equilibrium sample have a lower likelihood than expected, but which remains well under the intrinsic variations of likelihood of a sample of $P^{AR}$. We therefore conclude that even if our propagator has the undesirable property of going out of equilibrium at intermediate times, these deviations remain quite small.

### Branch Length Inference

To perform ancestral sequence reconstruction, not only the topology of the tree but also the branch lengths are needed. When comparing the autoregressive method to IQ-TREE, it would be unfair to use the branch lengths of the real tree since they do not correspond to the dynamical models used in IQ-TREE. For the same reason, using the branch lengths reconstructed by IQ-TREE would also be problematic. We thus perform reconstruction with the autoregressive by taking the tree inferred by IQ-TREE as an input and by reoptimizing its branches.

While optimizing branch lengths of a fixed topology is possible using site independent models, it is more challenging with the autoregressive evolver as it requires an explicit summation over all states at given internal nodes. For this reason, we resort to using a profile model with a shared substitution rate for this task. The algorithm used to reinfer branches is described in supplementary material Section A3, Supplementary Material online. In short, it attempts to scale the branches of IQ-TREE’s tree using a profile model. Supplementary fig. S4, Supplementary Material online shows the good quality of the reconstruction using this technique.

### Simulations

A simulation is performed as follows. First, a random tree of n=100 leaves is generated from Yule’s coalescent. We then normalize its height to a fixed value *H* that depends on the evolver model used: for the autoregressive model, we use H=2.0, while for the Potts model combined with Metropolis steps, we use H=8 sweeps, i.e. H=8L Metropolis steps where *L* is the length of the sequences.

A root sequence is sampled from the evolver model’s equilibrium distribution, and evolution is simulated along each branch independently starting from the root. In the case of the autoregressive evolve, the dynamics is the one of Equation (5). In the case of the Potts model, we use a Markov chain with the Metropolis update rule. In this way, we obtain for each repetition a tree and the alignments for internal and leaf nodes. Results presented in this work are obtained by averaging over M=100 such simulations for each protein family.

### Experimental Evolution Data

To validate the proposed method, we use data from Directed Evolution experiment on Beta-lactamase PSE-1 published in Stiffler et al. (2020). Beta-lactamase is an enzyme produced by bacteria that provides them resistance to the beta-lactam antibiotic class. Its activity relies on the ability to hydrolize the beta-lactam ring, inhibiting the effect of these antibiotics. In Stiffler et al. (2020), the PSE-1 wild-type (WT) undergoes 20 rounds of controlled in vivo evolution with an average target mutation rate of approximately 3%–4% per round while being selected for its inhibition effect on ampicillin. The bacterial population in the experiment is approximately $5\times 10^{4}$, and the fraction of bacteria surviving each selection round is around 1%. At round 20, the last one of the experiment, the library of mutated variants has accumulated an average Hamming distance from WT of 12.9% and an average pairwise distance of 19.8%.

A family of 42 k homologous sequences is available from PFAM with code PF13354. For this family, an Hidden Markov Model (HMM) of length 214, built on 66 seed sequences, is contextually available. We aligned the experimental sequences to the family HMM according to the following procedure:

1. the WT sequence (length 266) is aligned to the HMM using HMMER (Eddy 2023);
2. insertion sites in the aligned WT sequence are removed from the aligned WT sequence and from all the other sequences of the experimental library;
3. at positions where the aligned WT has a gap, a gap is also inserted in sequences of the experimental library.

This method ensures that all sequences from the experiment are aligned in the same manner.

It has been noticed in Bisardi et al. (2021) that taking into account the transition possibilities between amino acids allowed by the genetic code is important when describing short term evolutionary dynamics with generative models. In our framework, a natural way to include these is by using the symmetric matrix H in the decomposition of Equation (1). Terms of the H matrix do not affect the equilibrium distribution of the model, which thus remains generative, but influences the short term dynamics. Here, we simply counted the number of possibilities to transition from any amino acid to any other based on the genetic code, and we constructed the corresponding H matrix. The diagonal matrix remains given by the equilibrium probabilities of amino acids in the context of the sequence, as given by Equation (6). We found that this substantially improves the results of the autoregressive reconstruction for the experimental evolution data.

### Reconstruction with IQ-TREE

We run IQ-TREE using the -asr flag to generate states at internal nodes of the tree. By default, IQ-TREE reconstructs the maximum a posteriori (MAP) sequence at internal nodes (Yang et al. 1995). It also generates a “state” file containing the posterior probabilities of amino acids at each internal node that we use to sample internal sequences.

On simulated data, we ran IQ-TREE using the model finder routine to select the evolutionary model (Kalyaanamoorthy et al. 2017). For each simulated data set, i.e. a protein family and an evolver, we ran the model finder on a reduced set of trees. Since running the model finder is time consuming, we used these test runs to select a best model for each family/evolver and performed more extensive simulations using this one. The selected best models are reported in Table 1.

The model most frequently found was based on the PMB matrix (Veerassamy et al. 2003), with different options for rates depending on the family and evolver, e.g. +G4, +I+G4, or +R4 On the directed evolution data, the two most frequently found models were JTT (Jones et al. 1992) and a between patient HIV model (Nickle et al. 2007). Since the latter is clearly unrelated to the protein that is considered here, we used the JTT+G4 model for reconstruction.

In addition, we used IQ-TREE to perform reconstruction with profile mixture models, using the +C60 flag. Experiments with less complex models, e.g. +C10 and +C20, did not lead to an improvement as large as the +C60 flag: for this reason, we only show results for the latter. For each family, reconstruction was then performed using the model in Table 1 and appending the profile flag (e.g. legend of supplementary fig. S7, Supplementary Material online).

## Supplementary Material

msaf070_Supplementary_Data

## Data Availability

The code used in this work is accessible at the following links: the implementation of the reconstruction algorithm described here is available at https://github.com/PierreBarrat/AncestralSequenceReconstruction.jl the code used in simulations and data analysis is available at https://github.com/PierreBarrat/AutoRegressiveASR.
