# Supplementary material for: Reconstruction of Ancestral Protein Sequences Using Autoregressive Generative Models
Source: Mol Biol Evol. 2025 Mar 26;42(4):msaf070. doi: 10.1093/molbev/msaf070 (PMC12006719; doi:10.1093/molbev/msaf070)
Supplement: msaf070_Supplementary_Data [file msaf070_supplementary_data.zip › SI_standalone.pdf]

Supplementary Material: Reconstruction of ancestral protein sequences using autoregressive generative models

Matteo De Leonardis, Andrea Pagnani, Pierre Barrat-Charlaix

I. RECONSTRUCTION ALGORITHM

The classical pruning algorithm described in [1] allows one to compute, for each sequence position, the likelihood of the data at the leaves of a tree given an amino acid state at its root. It is then possible to infer marginal ancestral state by iteratively re-rooting the tree at all internal nodes and *e.g.* maximizing the corresponding posterior distribution of the root state. This technique is only possible if the model of evolution is reversible, in which case the position of the root is purely conventional.

Because the autoregressive model of evolution is irreversible, we cannot change the root of the tree and need to adapt the above algorithm. Our method is essentially an adaptation of the algorithm described in [2]. We first describe a general version of the algorithm, which could be used for any evolutionary model. We then explain how we apply it to our specific autoregressive evolver

A. General description of the algorithm

Our aim is to obtain, for each sequence position, a *marginal* reconstruction at each internal node. Given a node n in a rooted tree $\mathcal{T}$, calling x_n its amino acid state and $\mathcal{D}$ the amino acid states at the leaves, we want to compute the probability

$$\mathcal{L}_n(x) \stackrel{\text{def}}{\equiv} P(\mathcal{D}|\mathcal{T}, x_n = x), \tag{1}$$

that is the probability of the data knowing that n is in state x. We will see below that our way to compute $\mathcal{L}_n$ involves a prior distribution of internal states coming from the root node, and $\mathcal{L}_n$ is thus not strictly speaking a likelihood. However, we will abusively refer to it as likelihood in what follows. We define the maximum a posteriori (MAP) reconstruction as

$\arg\max_x \mathcal{L}_n(x)$, and a "posterior sampling" reconstruction as a sample from a normalized $\mathcal{L}_n(x)$. Note that since we consider a known and fixed tree and to lighten notation, we ignore the dependence on $\mathcal{T}$ in the following equations.

To compute $\mathcal{L}_n$, we introduce the following notation: let a be the ancestral and $\mathcal{C}_n$ the children nodes of n. Then, let $T_n(y,x)$ be the transition probability from amino acid state y to x for the branch $a \to n$. Importantly, T_n is a "directed" quantity: it describes the evolution from a to n. This is irrelevant for reversible models, but is important in the autoregressive case. Finally, we call q the number of different amino acid states that a site can be in: expressions of the form $\sum_{y=1}^{q}$ refer to sum over all amino acid states. In the autoregressive model, $q = 21$ for the 20 natural amino acids and the gap symbol.

First, we use the fact that if n is known to be in some state x, leaf-data on either sides of the branch $a \to n$ are independent. We call $\mathcal{D}_{below}$ the data at the leaves of the clade below n, and $\mathcal{D}_{above}$ the data at the leaves on the other side of the $a \to n$ branch. We can then write

$$\mathcal{L}_n(x) = P(\mathcal{D}_{below}|x_n = x)P(\mathcal{D}_{above}|x_n = x). \quad (2)$$

To simplify notation, we define the following quantities:

$$\begin{aligned} v_n(x) &= P(\mathcal{D}_{below}|x_n = x) \\ u_n(y) &= P(\mathcal{D}_{above}|y_a = y) \text{ where } a = \text{ancestor}(n) \end{aligned} \quad (3)$$

Note that $u_n(y)$ stands for the likelihood of $\mathcal{D}_{above}$ given that the *ancestor* a of n is in a given state y. This allows us to simplify Eq. 2 to obtain

$$\mathcal{L}_n(x) = v_n(x) \cdot \sum_{y=1}^{q} u_n(y) T_n(y,x). \quad (4)$$

In other words, we split the likelihood into a "below" term v depending on the state x of n, and an "above" term u depending on the state y of the ancestor a. The two are linked by the transition probability $T_n(y,x)$ along the branch $a \to n$. Summing over all states y then yields $\mathcal{L}_n(x)$.

To compute $v_n(x)$ and $u_n(x)$, we use the following set of recursive relations:

$$\begin{aligned} v_n(x) &= \prod_{c\in\mathcal{C}_n}\sum_{y=1}^{q} T_c(x,y)v_c(y) \\ &= \prod_{c\in\mathcal{C}_n}\left(\mathbf{T}_c\mathbf{v}_c\right)_x, \\ u_n(x) &= \sum_{y=1}^{q} u_a(y)T_a(y,x)\cdot\prod_{c\in\mathcal{C}_a\backslash n}\sum_{y=1}^{q} T_c(x,y)v_c(y) \\ &= \left(\mathbf{u}_a^T\mathbf{T}_a\right)_x\cdot\prod_{c\in\mathcal{C}_a\backslash n}\left(\mathbf{T}_c\mathbf{v}_c\right)_x, \end{aligned} \tag{5}$$

where we used bold-font symbols – *e.g.* $\mathbf{v}_n$ or $\mathbf{T}_n$ – to represent vector $[v_n(1),\dots,v_n(q)]$ and the $q\times q$ transition probability matrix $T(x,y)$.

The expression for $v_n(x)$ essentially says that the likelihood of data at the tips of the clade below n is a product of likelihoods coming from subclades of the children of n, each weighted by the transition matrix T_c of branch $n\to c$. On the other hand, the expression for $u_n(x)$ takes into account information coming from above the ancestor a – the term $\mathbf{u}_a^T\mathbf{T}_a$ – and from the children of a at the exception of n – the term $\prod_{c\in\mathcal{C}_a\backslash n}\mathbf{T}_c\mathbf{v}_c$. It is clear that fixing n, this set of recursive relations involves all leaves, and also all branches at the exception of the $a\to n$ one. This last branch is taken into account when combining $\mathbf{v}_n$ and $\mathbf{u}_n$ in Eq. 4. Finally, the set of relations is closed by the following conditions:

- if n is a leaf, $v_n(x)=\delta_{x,x_n}$ where δ is the Kronecker function and x_n the observed state at n.
- if n is the root, $u_n(x)=\pi(x)$ with $\pi=[\pi(1)\dots\pi(q)]$ being the equilibrium frequencies of amino acids according to the sequence evolution model.

Computing $\mathcal{L}_n(x)$ is done by applying the following steps.

- Traverse the tree in post-order and compute $\mathbf{v}_n$ for each node encountered. Since the traversal is post-order, $\mathbf{v}_c$ for $c\in\mathcal{C}_n$ is always available.
- Traverse the tree in pre-order and compute $\mathbf{u}_n$. Since the traversal is pre-order, $\mathbf{u}_a$ for $a=\text{ancestor}(n)$ is always known and $\mathbf{v}_n$ is known from the previous step.
- For each node n, compute $\mathcal{L}_n$ applying Eq. 4.

B. Application to the autoregressive model

Our autoregressive evolution model has the following unusual properties: *(i)* evolution depends on the relevant context, *e.g.* sites $1, \ldots, i-1$ for position i; *(ii)* as a corollary, the transition rate matrix Q defining evolution depends on the sequence *towards* which evolution is happening, as in Eq. 5; *(iii)* evolution is not reversible, meaning that the orientation of the branches of the tree matters.

We show below that the algorithm described above adapts without problems to these particularies. Reconstruction with the autoregressive model proceeds iteratively from the first to the last sequence position. Assume that we are reconstructing internal states at position i, and that positions $1, \ldots, i-1$ are already reconstructed for all internal nodes. We then apply the following steps.

- For all nodes n, compute the profile $\pi_{n,i}(x) = p_i(x|x_n^1, \ldots, x_n^{i-1})$, where p_i is a parameter of the autoregressive model defined in Eq. 4 and $x_n^1, \ldots, x_n^{i-1}$ is the context at node n.

- For all nodes n and given the equilibrium frequencies $\pi_{n,i}$ at this node and position, compute the transition probability matrix $\mathbf{T}_n$ for the branch ancestor(n) $\rightarrow n$. This matrix is defined as

$$\mathbf{T}_n = e^{t_n Q},$$

  with Q defined in Eq. 1 and t_n the length of the branch.

- When all transition matrices and node-specific equilibrium frequencies are known, apply the algorithm of the previous section to reconstruct state x_n^i at all nodes n.

C. Branch length inference

To reconstruct the branch length, we start from expressions of the likelihood Eq. 1 & Eq. 4. We first note that this expression is specific to a given sequence position $i \in \{1 \ldots L\}$, and thus rename quantities such as $\mathcal{L}_n$ to $\mathcal{L}_n^i$. Then, by summing over all possible states of internal node n, we obtain an expression for the probability of the data $\mathcal{D}_i$ at position i

knowing the tree:

$$\begin{aligned} P(\mathcal{D}_i|\mathcal{T}) &= \sum_{x=1}^{q} P(\mathcal{D}_i|\mathcal{T}, x_n = x) \\ &= \sum_{x=1}^{q} \mathcal{L}_n^i(x) \\ &= \sum_{x,y} u_n^i(y) T_n^i(y,x) v_n^i(x) \\ &= \left\langle \mathbf{u}_n^i | \mathbf{T}_n^i | \mathbf{v}_n^i \right\rangle . \end{aligned} \tag{6}$$

Finally, the likelihood of the all the leaf sequences is obtained by multiplying over sequence positions:

$$P(\mathcal{D}|\mathcal{T}) = \prod_{i=1}^{L} \left\langle \mathbf{u}_n^i | \mathbf{T}_n^i | \mathbf{v}_n^i \right\rangle . \tag{7}$$

Starting from this last expression, we use two techniques to infer MAP branch lengths. In practice, due to computational time considerations, we use the second one (branch scaling). Importantly, since Eq. 7 involves a product over all sequence positions, it is not possible to apply it to the autoregressive evolution model. Indeed, the only way to compute *e.g.* $\mathbf{v}_n^i$ for the autoregressive model is to have *fixed* the internal node states at positions $1, \ldots, i-1$, making $\mathbf{v}_n^1, \ldots, \mathbf{v}_n^{i-1}$ irrelevant. To avoid this difficulty, we apply the two methods below using a profile model with site specific frequencies instead of the autoregressive one.

1. *Single branch length optimization*

Expression Eq. 7 is practical because it allows one to compute the probability of the data as an explicit function of the transition matrices $\mathbf{T}_n^i$ of branch above node n ($\mathbf{v}_n$ and $\mathbf{u}_n$ do not depend on the branch above n). Note that since $\mathbf{T}_n^i = e^{t_n \mathbf{Q}_n^i}$, the dependence on the branch length t_n is also explicit. We use this to find the t_n that maximizes $P(\mathcal{D}|\mathcal{T})$:

$$t_n = \arg\max \sum_{i=1}^{L} \log \left\langle \mathbf{u}_n^i | e^{t_n \mathbf{Q}_n^i} | \mathbf{v}_n^i \right\rangle , \tag{8}$$

where we take the logarithm for computational reasons.

It is straightforward to obtain an analytical expression for the gradient of the above expression with respect to t_n, making optimization reasonably fast. We then optimize all

branch lengths starting from the IQ-TREE inferred tree and cycling over the following steps until convergence is reached:

- Compute messages $\mathbf{u}_n$ and $\mathbf{v}_n$ for all internal nodes n.
- Pick a non-root internal node n, and optimize its branch length t_n.

This algorithm is guaranteed to converge since the likelihood increases at each step. However, it is also computationally expensive: optimizing a single branch n requires computing the quantities $\mathbf{u}_n$ and $\mathbf{v}_n$, which in turn requires using the recursive relations in Eq. 5 over the whole tree. Since we assess the quality of ancestral reconstruction by applying it to many trees, we use in practice the quicker method described below

2. Scaling branch lengths

In order to make the branch length inference faster, we adopt a scaling strategy. We start from the tree inferred by IQ-TREE, using the settings described in the Methods section: for each node n, let t_n^0 be the branch length inferred by IQ-TREE. We construct the scaled tree $\mathbf{T}_\mu$ by multiplying the branches by a factor μ: the branch above any node n is $t_n = \mu t_n^0$. We then find the scaling factor μ that maximizes the likelihood:

$$\mu^\star = \arg\max_\mu P(\mathcal{D}|\mathcal{T}_\mu), \tag{9}$$

where the right-hand side can be numerically evaluated using the expression 7 at any internal node n (in our case, we use the root node). In contrast with the individual branch optimization, it is not possible to write the gradient of the likelihood with respect to μ, and we must use a derivative free optimization technique [3, 4]. However, since only one parameter must be optimized, this technique turns out to be much quicker for the trees of a hundred leaves that we use in the main text. The results can be seen in Figure 4.

II. AUTOREGRESSIVE EVOLUTION MODEL

A. Simplified expression for a homogeneous H

For each site i, the main difference between our model and a traiditional GTR is that the equilibrium frequencies of the Markov chain are computed using the context at the

previous sites $1, \ldots, i-1$. Considering Eq. 1 and Eq. 6, this means that the diagonal matrix is determined using the generative model. On the other hand, the symetric matrix $\mathbf{H}$ can be given any value without changing the long term generative properties of the dynamical model, *i.e.* Eq. 7. Here, we show that if the transitions defined by $\mathbf{H}$ are uniform, *i.e.* $H_{ab} = \mu$ for any $a \neq b$, the propagator takes a simplified form:

$$\begin{aligned} q_i(b_i|a_i, b_{<i}, t) &= e^{-\mu t}\delta_{a_i,b_i} + (1 - e^{-\mu t})p_i(b_i|b_{<i}), \\ P(\mathbf{b}|\mathbf{a}, t) &= \prod_{i=1}^{L} q_i(b_i|a_i, b_{<i}, t). \end{aligned} \tag{10}$$

The interpretation of the site propagator $q_i(b_i|a_i, b_{<i}, t)$ is straightforward: if no mutation occurs with probability $e^{-\mu t}$, site i remains in its original state a_i; otherwise, with probability $(1 - e^{-\mu t})$, it is resampled using the equilibrium probability given by the generative model and the context of the sequence $p_i(b_i|b_{<i})$. Note that the assumption of a scalar matrix is reasonable if one wishes to ignore the different transition rates between amino-acids. Interestingly, this form is analogous to the F81 model of DNA evolution [1], which also parametrizes the transition rate matrix $\mathbf{Q}$ using only the long term equilibrium frequencies $(\pi_A, \pi_C, \pi_G, \pi_T)$.

To lighten notation, we drop the explicit dependence on the position i and the sequence context $b_{<i}$ by defining $p_b = p_i(b_i|b_{<i})$. We will then compute the n eigenvectors and eigenvalues of $\mathbf{Q}$, where $n = 21$ for the amino acids and gap symbol. First, note that for the continuous time Markov chain to be well defined, we need the rows of $\mathbf{Q}$ to sum to 0. We thus have the following expression for the elements of $\mathbf{Q}$:

$$\mathbf{Q} = \mu \begin{pmatrix} p_1 - 1 & p_2 & \ldots & p_n \\ p_1 & p_2 - 1 & \ldots & p_n \\ \ldots & \ldots & \ldots & \ldots \\ p_1 & p_2 & \ldots & p_n - 1 \end{pmatrix} = \mu \left(\mathbf{1}\mathbf{p}^\dagger - I \right)$$

where $\mathbf{1}$ is the n-dimensional vector whose entries are all 1s, I is the identity matrix, and $\mathbf{p} = (p_1, \ldots, p_q)$. In particular we note that the outer product $\mathbf{1}\mathbf{p}^\dagger$ is a rank-one projector onto the state $\mathbf{p}$, and thus it has a left eigenvector equal to $\mathbf{p}^\dagger$ (associated to the eigenvalue 1) and $n-1$ eigenvalues equal to 0. Indeed:

$$\mathbf{p}^\dagger \mathbf{Q} = \mu \mathbf{p}^\dagger \left(\mathbf{1}\mathbf{p}^\dagger - I \right) = 0$$

As $p(b|a,t) = [\exp(\mathbf{Q}t)]_{ab}$, we need to compute the exponential of $\mathbf{Q}$. To do so, we first note that:

$$
\begin{aligned}
\mathbf{Q}^2 &= \mu^2 \left(\mathbf{1}\mathbf{p}^\dagger - I\right)\left(\mathbf{1}\mathbf{p}^\dagger - I\right) \\
&= \mu^2 \left(\mathbf{1}\underbrace{\mathbf{p}^\dagger\mathbf{1}}_{=1}\mathbf{p}^\dagger - 2\mathbf{1}\mathbf{p}^\dagger + I\right) \\
&= \mu^2 \left(-\mathbf{1}\mathbf{p}^\dagger + I\right) \\
&= -\mu\mathbf{Q}
\end{aligned}
$$

which in turn implies that $\mathbf{Q}^k = (-1)^{k-1}\mu^{k-1}\mathbf{Q}$. From this simple relation for all integer powers of $\mathbf{Q}$ we can explicitly compute the exponential of the $\mathbf{Q}$ matrix from following power series:

$$
\begin{aligned}
e^{t\mathbf{Q}} &= \sum_{k=0}^{\infty} \frac{t^k\mathbf{Q}^k}{k!} \\
&= I + \sum_{k=1}^{\infty} \frac{t^k\mathbf{Q}^k}{k!} \\
&= I - \frac{1}{\mu}\mathbf{Q}\sum_{k=1}^{\infty} \frac{t^k\mu^k(-1)^k}{k!} \\
&= I - \frac{1}{\mu}\mathbf{Q}\left(e^{-\mu t} - 1\right) \\
&= Ie^{-\mu t} + \mathbf{1}\mathbf{p}^\dagger\left(1 - e^{-\mu t}\right)
\end{aligned}
$$

We thus obtain the desired result:

$$
q(b|a,t) = e^{-\mu t}\delta_{ab} + (1 - e^{-\mu t})p_b. \tag{11}
$$

B. Non-Markovian and approximative nature of the propagator

The propagator of the main text is useful because it allows calculation of the transition *probability* between any two sequences and for any time. However, it is only an approximation, in a way that we show below. The structure of the next four paragraphs is as follows.

a. Our propagator does not respect global balance. The consequences are that *(i)* our dynamics is not Markovian and *(ii)* the generative model distribution P^{AR} is not stationary.

b. A consequence of the first point is that our propagator is irreversible.

c. Our propagator can be seen as an approximation of a continuous Markovian dynamic with P^{AR} as a stationary distribution. The approximation is exact at large times and at order one for small times.

d. The deviations between our approximate dynamics and the "correct" ones remain small for intermediate times.

The calculations below are valid for the simplified expression of the propagator in Eq. 10, that is for a uniform $\mathbf{H}$ in Eq. 1 of the main text. However, there is little doubt that the results are also valid for a more general $\mathbf{H}$. To simplify notation, we also consider the case $\mu = 1$: the case of a generic μ is easily re-derived.

1. *Non-Markovian nature*

A Markov chain that has a stationary distribution $\pi(\mathbf{a})$ and a transition probability matrix $q(\mathbf{b}|\mathbf{a})$ will verify *global balance*:

$$\pi(\mathbf{a}) = \sum_{\mathbf{b}} \pi(\mathbf{b}) q(\mathbf{a}|\mathbf{b}). \tag{12}$$

Here, we design a small toy example to show that our propagator does not in general satisfy global balance.

Consider a sequence of length $L = 2$ where each position can be in two states, 0 or 1. Assume that the "fitness landscape" of this protein is such that sequences $\{0,0\}$ and $\{1,1\}$ are equally functional, while $\{0,1\}$ and $\{1,0\}$ are not functional. Since an organism possessing sequences $\{0,1\}$ or $\{1,0\}$ would suffer a fitness loss, they would appear less frequently in nature. The sequence alignment of this "family" could then have the following statistics:

$$P(\{0,0\}) = P(\{1,1\}) = \frac{1}{2}(1-\varepsilon) \quad \text{and} \quad P(\{0,1\}) = P(\{1,0\}) = \frac{\varepsilon}{2}, \tag{13}$$

with $\varepsilon \ll 1$. A well-trained autoregressive model would consequently have the following properties:

$$\begin{aligned} &p_1(0) = p_1(1) = \frac{1}{2}, \\ &p_2(0|0) = p_2(1|1) = 1 - \varepsilon \quad \text{and} \quad p_2(0|1) = p_2(1|0) = \varepsilon. \end{aligned}$$

Indeed, state 0 or 1 are equally likely at position one, and given state a at position one the state at position two must also be a with probability $1-\varepsilon$. The corresponding autoregressive distribution P^{AR} is exactly equal to the natural one in Eq. 13.

We now set out to show that global balance does not hold in this case. Consider sequence $\{1,0\}$, which has probability $\varepsilon/2$. Then for any given time t we expect

$$P^{AR}(\{1,0\}) = \frac{\varepsilon}{2} = \sum_{\mathbf{a}} P^{AR}(\mathbf{a}) P(\{1,0\}|\mathbf{a},t),$$

where P is the propagator of Eq. 10.

To show the inequality, it is enough to consider one term of the sum on the right-hand side: the one with $\mathbf{a}=\{0,0\}$. Indeed, using Eq. 10 we immediately obtain

$$\begin{aligned} P^{AR}(\{0,0\}) P\left(\{1,0\}|\{0,0\},t\right) &= \frac{1-\varepsilon}{2} \cdot (1-e^{-t}) \frac{1}{2} \cdot \left(e^{-t} + (1-e^{-t})\varepsilon\right) \\ &\sim \mathcal{O}(1). \end{aligned}$$

Since at least one term in the sum is of order one and the terms are all positive, the sum itself is $\mathcal{O}(1)$. Since the left-hand side has order ε and ε can be chosen arbitrarily small, global balance cannot hold. Therefore, the target distribution P^{AR} of the autoregressive model, defined in Eq. 4 of the main text, is *not* the equilibrium of the propagator $P(\mathbf{b}|\mathbf{a},t)$ defined in Eq. 5.

Another important consequence is that the process is not Markovian. We know from the main text that at long times, $P(\mathbf{b}|\mathbf{a},t)$ converges to $P^{AR}(\mathbf{b})$. Injecting this in Eq. 12, we see that that global balance holds for $t\to\infty$. If $P(\mathbf{b}|\mathbf{a},t)$ was a Markov process, this would mean that P^{AR} is its stationary distribution and that global balance should hold at all times t. As the example above shows, this is not the case. Therefore, our process is not Markovian.

2. Irreversibility

For a stochastic model with stationary distribution π and transition probability $q(\mathbf{b}|\mathbf{a},t)$, time reversibility is equivalent to respecting *detailed balance*: for any two sequences $\mathbf{a}$ and $\mathbf{b}$ and any time t, one should have

$$\pi(\mathbf{a}) q(\mathbf{b}|\mathbf{a},t) = \pi(\mathbf{b}) q(\mathbf{a}|\mathbf{b},t). \tag{14}$$

Detailed balance implies global balance, as summing over either $\mathbf{a}$ or $\mathbf{b}$ in Eq. 14 directly gives Eq. 12. As the previous section showed, the autoregressive propagator does not satisfy global

balance. Therefore, it cannot be time reversible. We stress that the cause of irreversibility here is not epistasis in itself, but rather the structure of the autoregressive propagator. In fact, it is perfectly possible to design dynamical epistatic models that are time reversible, either with discrete time [5] or with continuous time (Section II B 3).

Note that irreversibility only happens at the sequence level, and not for individual positions. Indeed for each position i and given a sequence context, the autoregressive model has the same structure as classical sequence evolution models. In particular, it is time reversible: given a context and any two amino acid states a_i and b_i, there is no objective way of determining whether a_i evolved in to b_i or the reverse.

3. Instantaneous transition rates

If the autoregressive propagator was Markovian, it would be defined by its transition rate matrix $\mathbf{Q}$:

$$P(\mathbf{b}|\mathbf{a},t) \sim \left(e^{t\mathbf{Q}}\right)_{\mathbf{ab}}, \tag{15}$$

where we use the $\sim$ symbol to remind that the above equation does not actually hold. Note that the $\mathbf{Q}$ here is a sequence-to-sequence transition rate matrix of dimension $q^L \times q^L$ where $q = 21$ is the number of amino-acid plus the gap symbol. It is different from the position specific Q^i of the main text.

As we have seen, the process is not Markovian. However, we can still calculate the instantaneous transition rate by defining

$$Q_{\mathbf{ab}} \overset{\text{def}}{\equiv} \left.\frac{\mathrm{d}P(\mathbf{b}|\mathbf{a},t)}{\mathrm{d}t}\right|_{t=0}. \tag{16}$$

Doing so in the case where $\mathbf{H}$ is uniform and using Eq. 10 for the transition probabilities yields the following $\mathbf{Q}$:

$$Q_{\mathbf{ab}} = \begin{cases} 0 \quad \text{if } \mathbf{a} \text{ and } \mathbf{b} \text{ differ at more than two sites,} \\ p_i(b_i|a_{<i}) \quad \text{if } \mathbf{a} \text{ and } \mathbf{b} \text{ differ only at site } i, \\ \sum_{i=1}^{L}(p_i(a_i|a_{<i}) - 1) \quad \text{if } \mathbf{a} = \mathbf{b}, \end{cases} \tag{17}$$

where the p_i are the conditional probabilities defined by the autoregressive model. This form is very similar to the one used in other works dealing with epistatic model in phylogenetics [6–8]. It is quite straightforward to interpret: the transition rate for sequences at distance

strictly higher than one vanishes, meaning that at most one substitution can occur in an infinitesimal amount of time; if two sequences differ at site i, then the transition rate is the probability of observing the new amino acid b_i in the context of the starting sequence $\mathbf{a}$. The diagonal elements ensure that lines of $\mathbf{Q}$ sum to 0.

It is interesting to note that the stationary distribution for $\mathbf{Q}$ is the generative distribution $P^{AR}(\mathbf{a}) = \prod_{i=1}^{L} p_i(a_i|a_{<i})$, that is:

$$\sum_{\mathbf{a}} P^{AR}(\mathbf{a}) Q_{\mathbf{ab}} = 0 \quad \text{for all sequences } \mathbf{b}. \tag{18}$$

To demonstrate this, we first note $\mathcal{N}_i(\mathbf{b})$ the ensemble of sequences that differ from $\mathbf{b}$ at position i only. Using Eq. 17, we can write

$$\begin{aligned}\sum_{\mathbf{a}} P^{AR}(\mathbf{a}) Q_{\mathbf{ab}} =& \sum_{i=1}^{L} \sum_{\mathbf{a}\in\mathcal{N}_i(\mathbf{b})} P^{AR}(\mathbf{a}) p_i(b_i|a_{<i}) + P^{AR}(\mathbf{a}) \sum_{i=1}^{L} (p_i(a_i|a_{<i}) - 1) \\ =& \sum_{i=1}^{L} \sum_{\mathbf{a}\in\mathcal{N}_i(\mathbf{b})} p_i(b_i|a_{<i}) \prod_{j=1}^{L} p_j(a_j|a_{<j}) + P^{AR}(\mathbf{b}) \sum_{i=1}^{L} (p_i(b_i|b_{<i}) - 1),\end{aligned}$$

where the first term involves all sequences at distance one from $\mathbf{b}$ and the second handles the case $\mathbf{a} = \mathbf{b}$. To make progress, we note that the sum over $\mathcal{N}_i(\mathbf{b})$ can be simplified as follows (for a generic function f):

$$\begin{aligned}\sum_{\mathbf{a}\in\mathcal{N}_i(\mathbf{b})} f(\mathbf{a}) =& \sum_{\mathbf{a}} \left(f(\mathbf{a}) \prod_{\substack{j=1 \\ j\neq i}}^{L} \delta_{a_j, b_j} \right) \\ =& \sum_{\substack{a_i=1 \\ a_i\neq b_i}}^{q} f(b_1, \ldots, b_{i-1}, a_i, b_{i+1}, \ldots, b_L).\end{aligned}$$

This essentially means that inside the sum the symbol a_j can be transformed into b_j if $j \neq i$, and that the remaining symbol a_i is traced over with the condition $a_i \neq b_i$. Using this, our

calculation yields

$$\begin{aligned}\sum_{\mathbf{a}} P^{AR}(\mathbf{a}) Q_{\mathbf{ab}} &= \sum_{i=1}^{L} p_i(b_i|b_{<i}) \prod_{\substack{j=1\\ j\neq i}}^{L} p_j(b_j|b_{<j}) \sum_{\substack{a_i=1\\ a_i\neq b_i}}^{q} p(a_i|b_{<i}) \\ &+ P^{AR}(\mathbf{b}) \sum_{i=1}^{L} (p_i(b_i|b_{<i}) - 1) \\ &= P^{AR}(\mathbf{b}) \sum_{i=1}^{L} (1 - p(b_i|b_{<i})) + P^{AR}(\mathbf{b}) \sum_{i=1}^{L} (p_i(b_i|b_{<i}) - 1) \\ &= 0.\end{aligned}$$

What this means is that the $\mathbf{Q}$ of Equations 16 and 17 is the one that we would like to use: it defines a time reversible Markov process with a stationary distribution P^{AR} that is generative. We call P' this "correct" Markov process, which is defined by

$$P'(\mathbf{b}|\mathbf{a}, t) = (e^{t\mathbf{Q}})_{\mathbf{ab}}. \tag{19}$$

However, since matrix $\mathbf{Q}$ is of dimensions $q^L \times q^L$ and we do not know how to compute its eigenvectors, we cannot actually compute $P'(\mathbf{b}|\mathbf{a}, t)$.

Instead we use the process P introduced in the main text, which has two properties: *(i)* its derivative at $t = 0$ is $\mathbf{Q}$ (Eq. 16) and *(ii)* it has P^{AR} as a stationary state for $t \to \infty$. In other words, P verifies the following:

$$\begin{aligned}&P(\mathbf{b}|\mathbf{a}, t) \simeq (\mathbb{1} + t\mathbf{Q})_{\mathbf{ab}} \simeq P'(\mathbf{b}|\mathbf{a}, t) \quad \text{for } t \ll 1, \\ &P(\mathbf{b}|\mathbf{a}, t) - P'(\mathbf{b}|\mathbf{a}, t) \xrightarrow[t\to\infty]{} 0,\end{aligned} \tag{20}$$

where $\mathbb{1}$ is the identity matrix. In other words, the propagator of the main text is an approximation of the continuous time dynamics associated with P^{AR}, which becomes exact at small and large times.

4. Deviations at intermediate times

An undesired consequence of our approximation is that when starting with sequences sampled from the target distribution P^{AR}, the propagator P of the main text generates out-of-equilibrium sequences at intermediate times. On the contrary, equilibrium would be maintained if using the exact propagator P' of Eq. 19. In mathematical terms, and using the

notation of the previous section, we would have

$$\begin{aligned}\sum_{\mathbf{a}} P^{AR}(\mathbf{a})P'(\mathbf{b}|\mathbf{a},t) &= P^{AR}(\mathbf{b})\\ \sum_{\mathbf{a}} P^{AR}(\mathbf{a})P(\mathbf{b}|\mathbf{a},t) &= \pi_t(\mathbf{b}),\end{aligned} \tag{21}$$

where π_t is a distribution over sequences that becomes equal to P^{AR} for $t \to 0$ and $t \to \infty$. In order to quantify how far from equilibrium the model goes, we need to compare P^{AR} and π_t at intermediate times. We do this by performing two numerical experiments.

First, starting from an initial sequence $\mathbf{a}$ sampled from P^{AR}, we compute the average log-likelihood of sequences sampled from $P(\mathbf{b}|\mathbf{a},t)$. We then average this process over $\mathbf{a}$ to define

$$\mathcal{L}(t) = \sum_{\mathbf{a},\mathbf{b}} P^{AR}(\mathbf{a})P(\mathbf{b}|\mathbf{a},t)\log\left(P^{AR}(\mathbf{b})\right) = \sum_{\mathbf{b}} \pi_t(\mathbf{b})\log\left(P^{AR}(\mathbf{b})\right). \tag{22}$$

For a perfect approximation, $\mathcal{L}(t)$ should remain equal to the average log-likelihood of sequences sampled from the generative model at all times. The right panel of Figure S1 shows that $\mathcal{L}(t)$ drops at intermediate times, which means that our propagator generated sequences that are "worse" than the generative model. However, the magnitude of this drop (about 5 at its minimum) is small when compared to the distribution of log-likelihoods sampled from P^{AR}. It is also small compared to the biases in the likelihood of reconstructed sequences shown in Figure 2 of the main text.

Our second test consists in using a tree generated in the same way as the ones used in the main text, and to simulate evolution using our autoregressive model by starting from an equilibrated root sequence. We then compute the distribution of log-likelihood of the leaves sequences. Again, for a process that is always at equilibrium, the distribution at the leaves should be the same as the one used to generate the root. The left panel of Figure S1 shows that this is not the case, with the log-likelihood of the leaves being on average lower. However, the two distributions are still quite close, in particular for their left tail.

We conclude from these experiments that even if our propagator has the undesirable property of going out of equilibrium at intermediate times, these deviations remain quite small. The autoregressive propagator can thus be seen as a useful *approximation*, allowing reconstruction at internal nodes without sacrificing much of the generative properties of the original model.

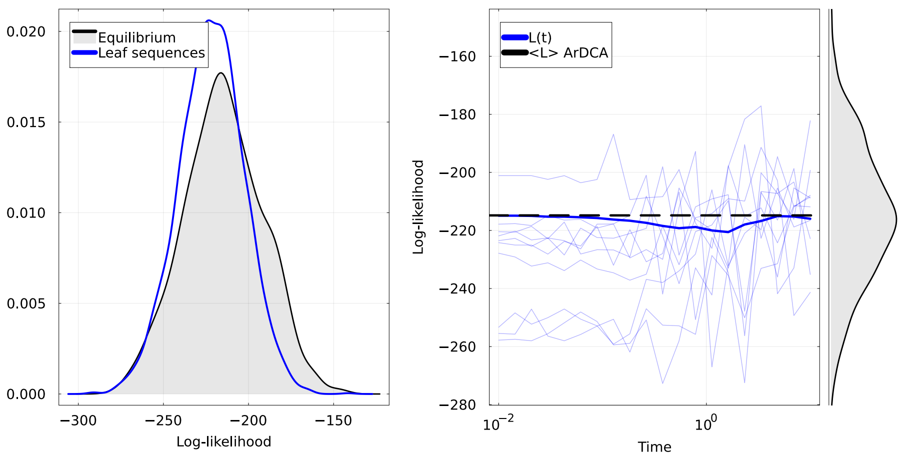

FIG. 1. Because it does not respect global balance, the propagator generates "out of equilibrium" sequences at intermediate times. **Left**: Distribution of log-likelihood of sequences at the tips of a tree (blue curve), when simulated using the autoregressive propagator and with a root sampled from the ArDCA model. For a dynamics that remains in equilibrium, the distribution should match the one of the ArDCA model (black curve). The shift indicates a slight out of equilibrium behavior. The tree used is generated in the same way as those used in the main text. **Right**: Log-likelihood of trajectories obtained by sampling the auto-regressive propagator at different times. Thin blue curves are example individual trajectories, with the initial sequence taken randomly from the equilibrium distribtion of the ArDCA model. The thick blue curve is the average of many individual trajectories. The black curve is the average log-likelihood of sequences sampled from the ArDCA equilibrium distribution. The drop in average likelihood around $t = 1$ is indicative of the out of equilibrium behavior. However its amplitude remains small with respect to the width of the equilibrium distribution

C. Position of the root

Because the autoregressive model is irreversible, the probability of a reconstruction depends on the orientation of the branches of the tree, and thus on the placement of the root. To quantify this dependence, we perform the following numerical experiment.

1. *Original tree and reconstruction.* We first generate a tree at random and simulate evolution on it using the autoregressive model, using the same procedure as in the main text. Note that by construction, the placement of the root for this original tree is

known exactly. We then perform ancestral reconstruction using the same autoregressive model, and refer to these ancestral sequences as the *original reconstruction*.

2. *Reconstruction on re-rooted trees.* We then iteratively root the original tree at each internal node, and perform reconstruction again using the same leaf sequences as before. In this case, the placement of the root is wrong in the sense that it does not correspond to the evolutionary process that generated the leaf sequences.

We use original trees of $n = 100$ leaves, and make 10 repetitions of this experiment. For a given repetition, the sequence at each internal node is reconstructed $n - 1 = 99$ times. Since there are 99 internal nodes and 10 repetitions, we obtain a set of $\sim 10^5$ reconstructed sequences. For each of these, we can compute:

- the amplitude of the re-rooting event, that is the branch-length distance between the original root of the tree and the one for which the reconstruction was performed;
- the variation with respect to the original reconstruction, measured in Hamming distance;
- the loss in performance, that is the increase in Hamming distance to the real ancestor with respect to the original reconstruction.

Figure S2 shows the results of this experiment. On its top-left panel, we see that there are indeed variations in the reconstructed sequences when changing the position of the root. However, the amplitude of these variations are quite limited, as they are on average smaller than 0.4%. We find the loss in performance to be one order of magnitude lower, typically around 0.05%. This suggests that the variations mostly occur at sites where the reconstruction was unreliable to begin with.

The top-right panel shows the same quantities but only for nodes that are close to the original root of the tree (distance < 0.1). These are nodes where we can expect more variation, as they are located far from the leaves. We indeed see that there reconstruction varies much more when the root is changed, with a difference of up to 0.08 Hamming distance extreme root misplacements. On the other hand, the loss in performance of the reconstruction remains very small, on the order of 0.1%. Again, this suggests that the change in reconstructed sequence when misplacing the root mostly occurs in parts of the sequence that were unreliable to begin with.

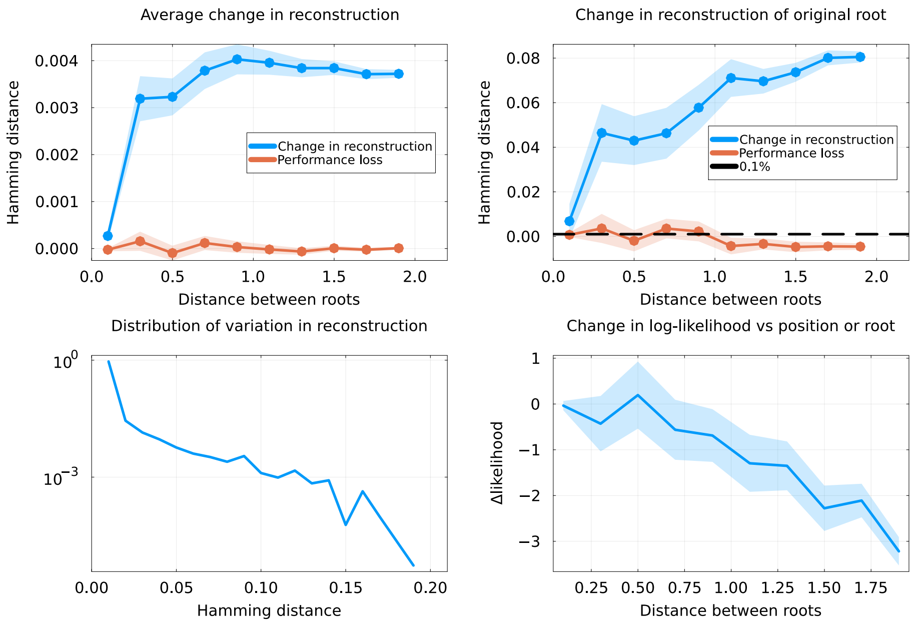

FIG. 2. Dependence of ancestral sequence reconstruction on the position of the root. **Top-left**: Variation in sequence reconstruction and loss of performance as a function of the amplitude of the re-rooting. The blue curve shows the average Hamming distance between MAP ancestral sequences when using the original tree (*i.e.* correct root placement) or a re-rooted tree, as a function of the amplitude of the re-rooting. The orange curve shows the degradation in reconstruction performance when changing the root position. **Top-right**: Same as top-left, but showing only nodes that are close (distance < 0.1) to the original root of the tree. These nodes are the farthest away from the leaves. Variation in the reconstruction is clearly larger, but the loss in performance remains very small. **Bottom-left**: Distribution of the variation in reconstruction for re-rooting of large amplitude (*i.e.* distance > 1.5): most reconstructions vary very little. In rare cases, the reconstruction varies significantly: in 0.2% of cases, the Hamming distance between two reconstructions is greater than 10%. **Bottom-right**: Average change in log-likelihood of the reconstruction of the root as a function of the amplitude of the re-rooting.

The bottom-left panel shows the distribution of variation in reconstruction for the larger root displacements (about 70 000 reconstructions). As expected, the variation is small in the vast majority of cases. Interestingly however, we observe that changing the root of the tree leads to large fluctuations in reconstruction in rare cases. For instance, in about 0.2% of cases, the Hamming distance between two reconstructions is greater than 10%.

Finally, the bottom-right shows that the likelihood of the reconstruction of the new root sequence decreases with how far it is placed from the original root. This means that if the position of the root was unknown, it could still be guessed with reasonable accuracy based on the likelihood.

III. DIRECTED EVOLUTION DATA

A. Minimum reconstruction error of the consensus

In the left panel of Figure 4, the Hamming distance of the consensus of M sequences to the wild-type sequence shows a minimum for an intermediate value of M. This is at first counter-intuitive, and we present here a minimalistic example to illustrate this phenomenon.

We consider the simplified case with binary sequences of length L and a star-like tree with M leaves at equal distance from the root. The root sequence is $\mathbf{r} = (0, \ldots, 0)$, and the sequence of leaf m is $\mathbf{x}^m = (x_1, \ldots, x_L)$ with $x_i \in \{0, 1\}$. We now assume that the first site in the sequence is much more variable than the others, so that it is frequent for sequence x^m to have a 1 at position $i = 1$, but rare at positions $i > 1$. The probability of observing state 1 at a site i in a leaf sequence is

$$P(x_i^m = 1) = \begin{cases} \frac{1}{2} + \varepsilon & \text{if } i = 1, \\ \varepsilon & \text{if } i \neq 1, \end{cases} \tag{23}$$

where $\varepsilon > 0$ is a parameter that in principle depends on the root-to-tip distance of the tree.

We now consider the consensus of the leaf-sequences and how close it is to the root $\mathbf{r} = (0, \ldots, 0)$. For the first position $i = 1$, the probability that the consensus differs from the root is the probability that more than $M/2$ leaves have mutated at this position. This is the probability that a binomial variable of parameters $\left(\frac{1}{2} + \varepsilon, M\right)$ takes a value larger than $M/2$: we call $\alpha\left(\frac{1}{2} + \varepsilon, M\right)$ this probability. Likewise, for a position $i > 1$, the probability that the consensus differs from the root is the probability $\alpha(\varepsilon, M)$ that a binomial variable of parameters (ε, M) takes a value larger than $M/2$.

It is then immediate that the average Hamming distance $H(M)$ between the consensus and the root if there are M leaves is

$$\langle H(M)\rangle = (L-1)\alpha(\varepsilon, M) + \alpha\left(\frac{1}{2}+\varepsilon, M\right). \tag{24}$$

Ideally, we would like to show that for certain values of ε, $\langle H(M)\rangle$ has a minimum at intermediate M. Unfortunately, we are unable to give analytical expressions for $\alpha(p, M)$ for generic p and M. Before exploring this with a numerical simulation, we show that in our setup the consensus of $M = 1$ sequence can be better than the consensus of an infinite number of sequences. The limits of α for large and small M are easily obtained:

$$\alpha(p, M = 1) = p \quad \text{and} \quad \alpha(p, M \to \infty) = \begin{cases} 1 & \text{if } p > \frac{1}{2} \\ 0 & \text{if } p < \frac{1}{2} \end{cases}.$$

Here, with $0 < \varepsilon < 1/2$, we have $\alpha\left(\frac{1}{2}+\varepsilon, M \to \infty\right) = 1$ and $\alpha(\varepsilon, M \to \infty) = 0$. In other words, for $M \to \infty$, the consensus at the first site will always differ from the root (as expected because it mutates "fast") while the consensus at other slow-evolving sites will be equal to the root state. We therefore obtain

$$\langle H(M \to \infty)\rangle = 1 \quad \text{and} \quad \langle H(M = 0)\rangle = L\varepsilon + \frac{1}{2}. \tag{25}$$

If $L\varepsilon < 1/2$, we observe that on average, the consensus of one sequence is closer to the root than the consensus of an infinite number of sequences.

The general case is explored in Figure S3: we show the numerical values of these $\alpha\left(\frac{1}{2}+\varepsilon, M\right)$ and $\alpha(\varepsilon, M)$ for $\varepsilon = 0.05$ and $L = 10$. The first term $\alpha\left(\frac{1}{2}+\varepsilon, M\right)$ increases monotonically from $\frac{1}{2}+\varepsilon$ to 1, while the second decreases from ε to 0. Combining the two with Eq. 24, we see that $\langle H(M)\rangle$ has a minimum at an intermediate M.

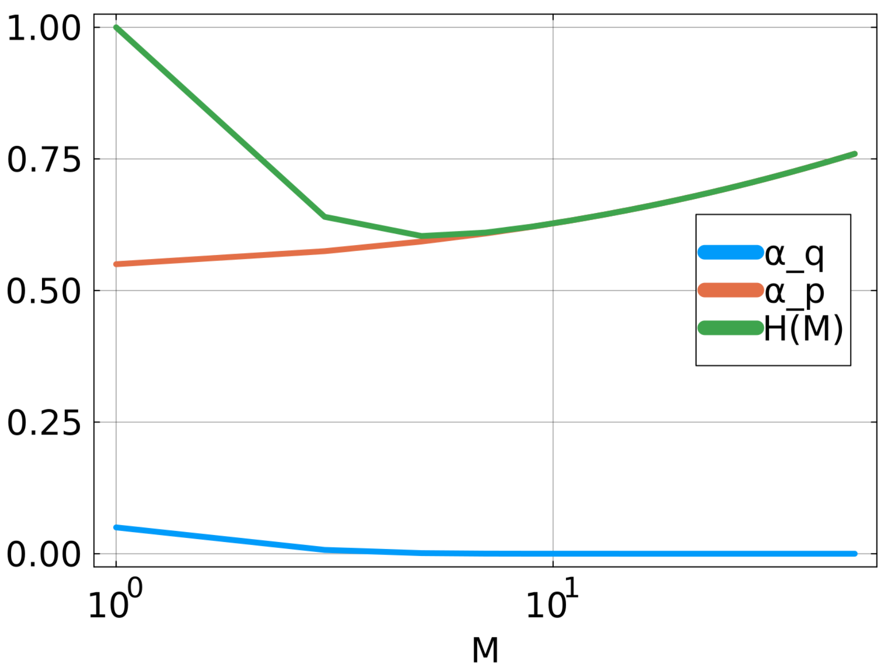

FIG. 3. Quantities $\alpha\left(\frac{1}{2}+\varepsilon, M\right)$, $\alpha\left(\varepsilon, M\right)$ and $\langle H\rangle$ as a function of the number of leaves M (odd values only). $\alpha(p, M)$ is defined to be the probability that a binomial variable of parameters (p, M) takes a value below $M/2$. $\alpha\left(\frac{1}{2}+\varepsilon, M\right)$ is increasing from $1/2+\varepsilon$ to 1 while $\alpha\left(\varepsilon, M\right)$ is decreasing from ε to 0. The average Hamming distance reaches a minimum for an intermediate number of leaves. Values of parameters: $\varepsilon = 0.05$, $L = 10$.

IV. SUPPLEMENTARY FIGURES

A. Extra figures

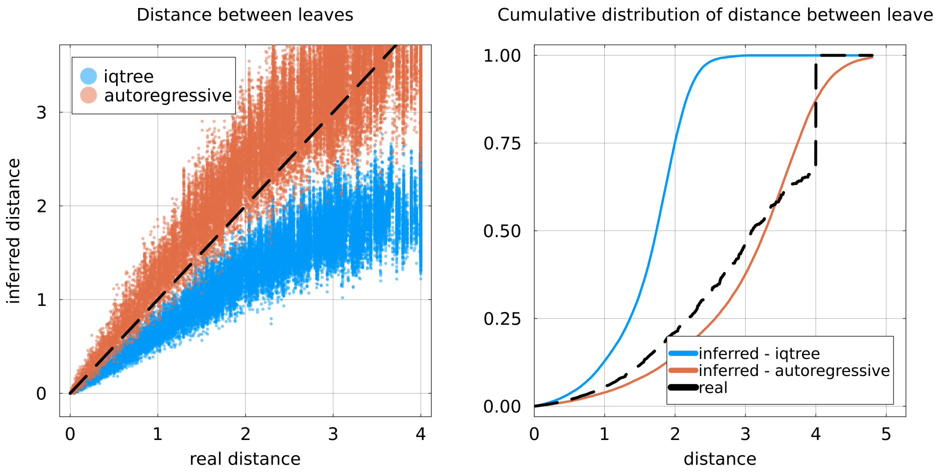

FIG. 4. Quality of branch length inference with the single-branch technique of section I C 2, using data simulated with the autoregressive evolver and a tree with fixed topology. This is the technique used in the reconstructions of the main text. The original branch lengths inferred by IQ-TREE are displayed for comparison. **Left**: inferred distance vs distance in the real trees for every pair of leaves. **Right**: Cumulative distribution of pairwise distance along the tree between leaves for the two inference methods and for the real tree. The discontinuity in the curve for the real tree is caused by the ultrametricity and fixed total height of the generated trees.

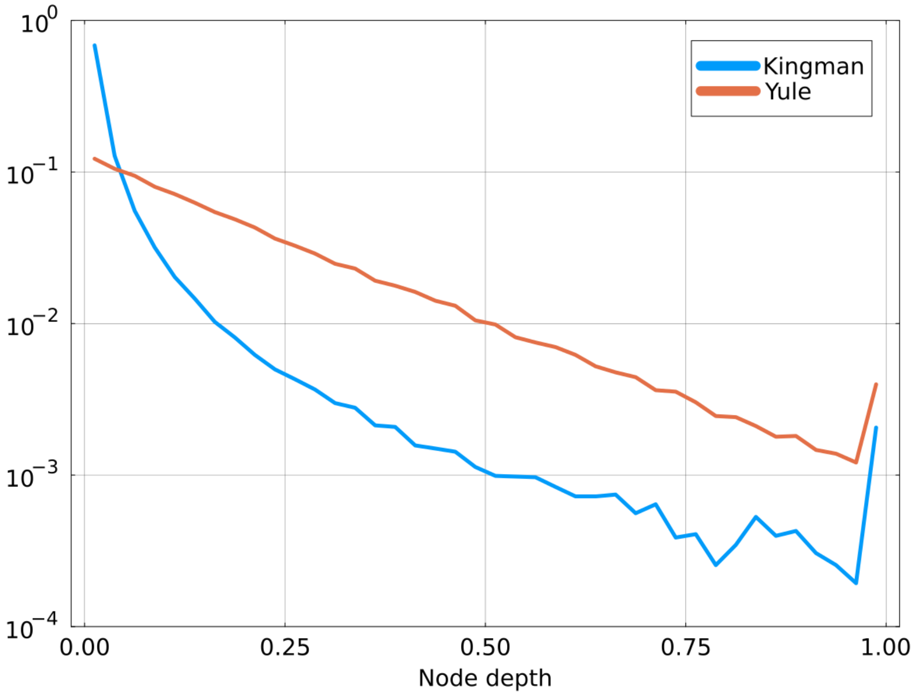

FIG. 5. Distribution of node depth for trees coming from the Kingman and Yule coalescents. Node depth is defined as the distance from a node to the closest leaf. Data is obtained by sampling several trees from each coalescent. Heights of trees are normalized to one. The Kingman process concentrates most of the nodes in close vicinity to the leaves, while the Yule process spreads them more evenly.

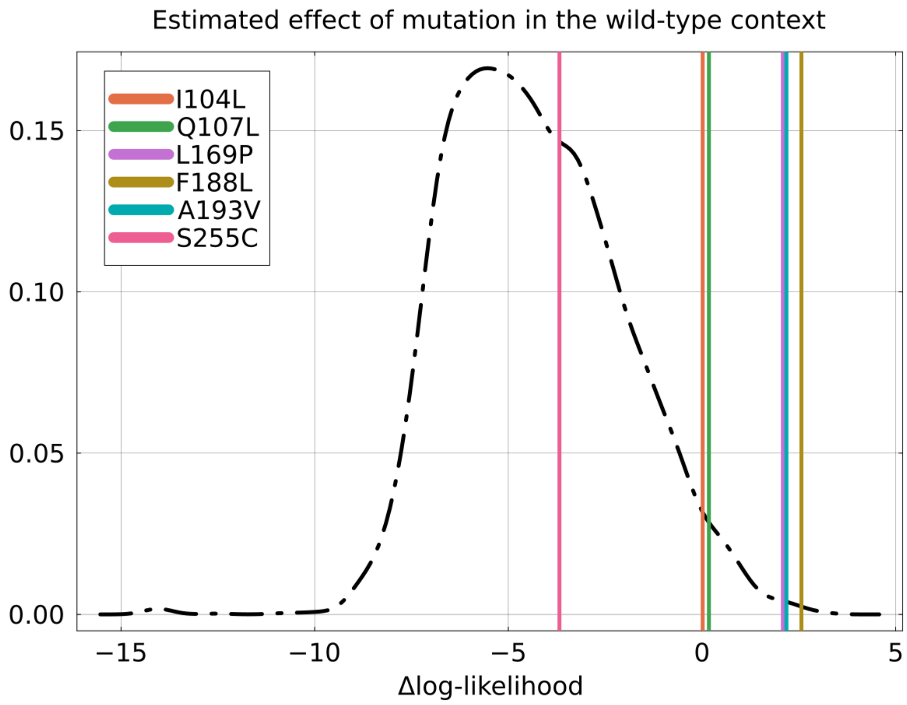

FIG. 6. Distribution of estimated effect of single mutations by ArDCA in the PSE1 sequence (black curve). The effect of a mutations is estimated by computing the difference in log-likelihood between the mutant sequence and the wild-type: negative values are detrimental and 0 represents a neutral mutation. As expected, most mutations are estimated to be detrimental but mutations found in the consensus of round 20 are mostly beneficial or neutral. The six reconstruction errors in Figure 4 are displayed as vertical bars. The two positions 169 and 193 where ArDCA outperforms IQ-TREE correspond to beneficial mutations.

B. Reconstruction of PF00072 using profile models

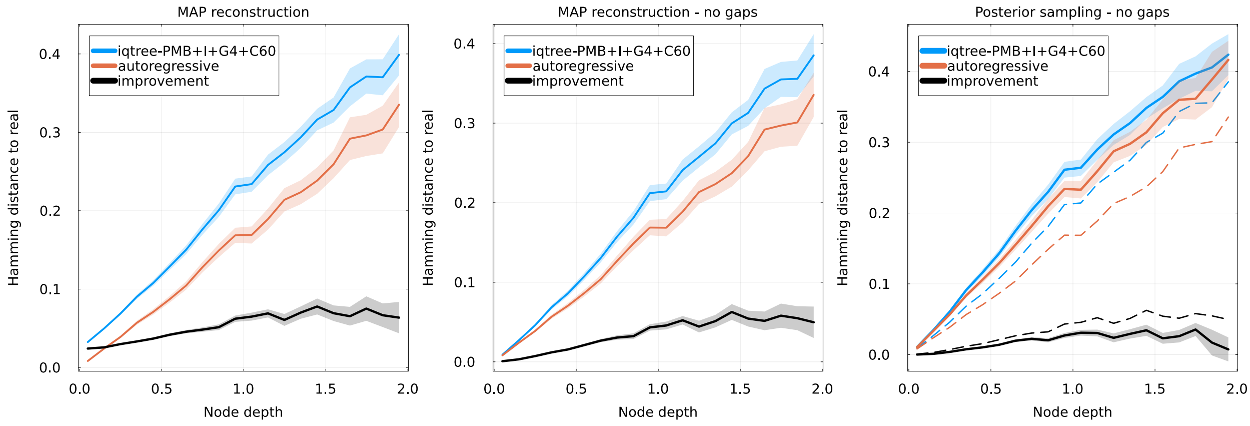

FIG. 7. Equivalent to Figure 1 of the main text, but using the `+C60` flag in IQ-TREE's reconstruction (profile model).

Hamming distance between reconstructed and real sequences as a function of node depth, using IQ-TREE and our autoregressive approach. The evolution model used by IQ-TREE is reported in the legend. The difference between the two methods ("improvement") is shown as a black curve. Estimation of the uncertainty is shown as a ribbon. The evolver and reconstruction autoregressive models are learned on the PF00072 family. **Left**: Hamming distance between the full aligned sequences, gaps included, using maximum a posteriori reconstruction. **Center**: Hamming distance ignoring gapped positions, using MAP reconstruction. **Right**: comparison of posterior sampling (solid lines) and MAP (dashed lines) reconstructions, ignoring gaps.

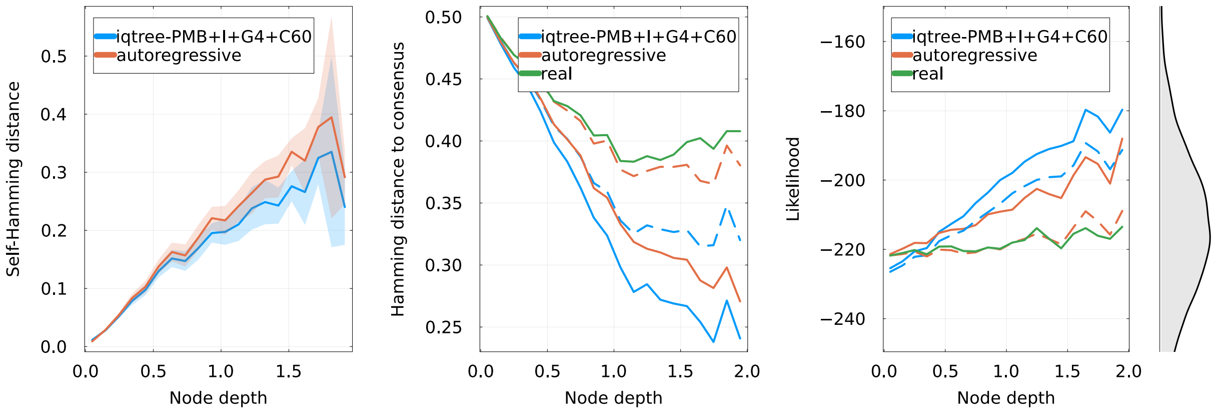

FIG. 8. Equivalent to Figure 2 of the main text, but using the `+C60` flag in IQ-TREE's reconstruction (profile model).

Left: for posterior sampling reconstruction, average pairwise Hamming distance among sequences reconstructed for each internal node. This quantifies the diversity of possible ancestral reconstructions. **Center**: Hamming distance between reconstructed sequences and the consensus sequence of the alignment. Solid lines represent MAP reconstruction or the real internal sequences, and dashed lines posterior sampling. IQ-TREE appears more biased towards the consensus sequence. **Right**: Log-likelihood of reconstructed and real sequences in the autoregressive model, *i.e.* using the logarithm of Eq. 4. MAP methods (orange and blue solid lines) are biased towards more probable sequences. Posterior sampling autoregressive reconstruction gives sequences that are at the same likelihood level than the real ancestors. The equilibrium distribution of likelihood of sequences generated by Eq. 4 is shown on the right.

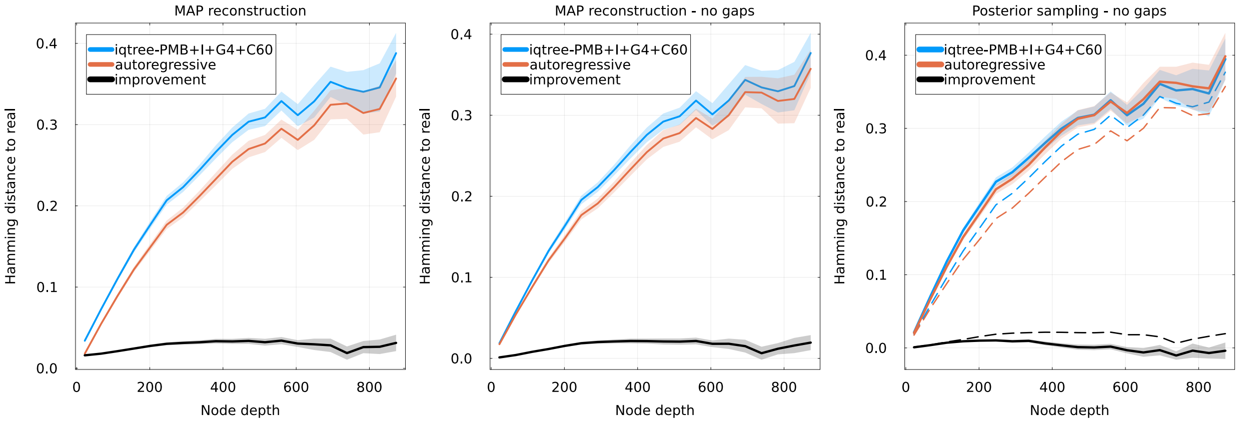

FIG. 9. Equivalent to Figure 3 of the main text. Analogous to Figure 7, but using a Potts model as the evolver. Hamming distance between reconstructed and real sequences as a function of node depth, using IQ-TREE and our autoregressive approach. The difference between the two methods is shown as a black curve. The evolver and reconstruction autoregressive models are learned on the PF00072 family. **Left**: Hamming distance between the full aligned sequences, gaps included, using MAP reconstruction. **Center**: Hamming distance ignoring gapped positions, using MAP reconstruction. **Right**: comparison of posterior sampling (solid lines) and MAP (dashed lines) reconstructions, ignoring gaps.

C. Results for other protein families

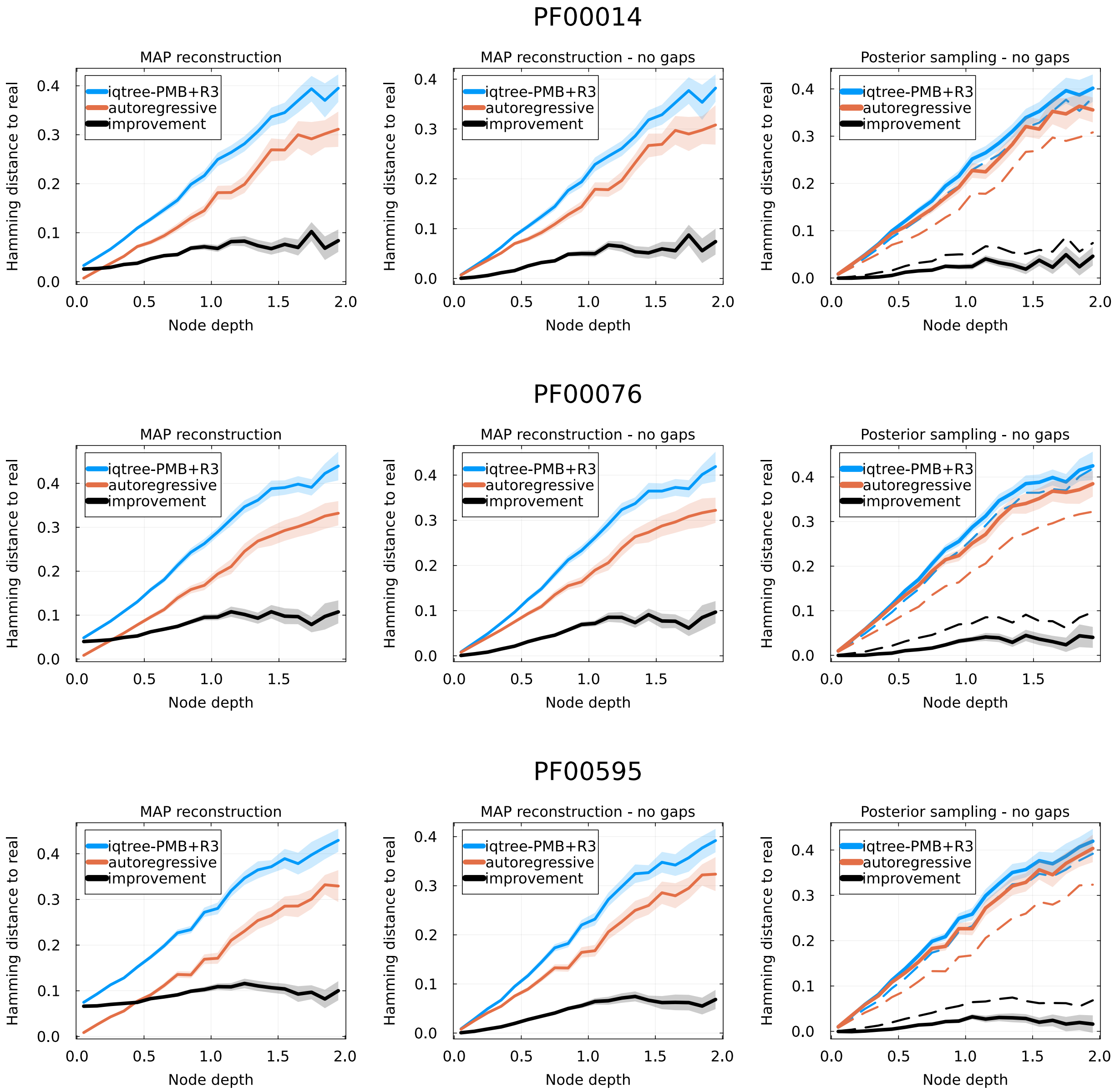

FIG. 10. Equivalent to Figure 1 of the main text using three other protein families. Hamming distance between reconstructed and real sequences as a function of node depth, using IQ-TREE and our autoregressive approach. The evolution model used by IQ-TREE is reported in the legend. The difference between the two methods ("improvement") is shown as a black curve. Estimation of the uncertainty is shown as a ribbon. The evolver and reconstruction autoregressive models are learned on the PF00072 family. **Left**: Hamming distance between the full aligned sequences, gaps included, using maximum a posteriori reconstruction. **Center**: Hamming distance ignoring gapped positions, using MAP reconstruction. **Right**: comparison of posterior sampling (solid lines) and MAP (dashed lines) reconstructions, ignoring gaps.

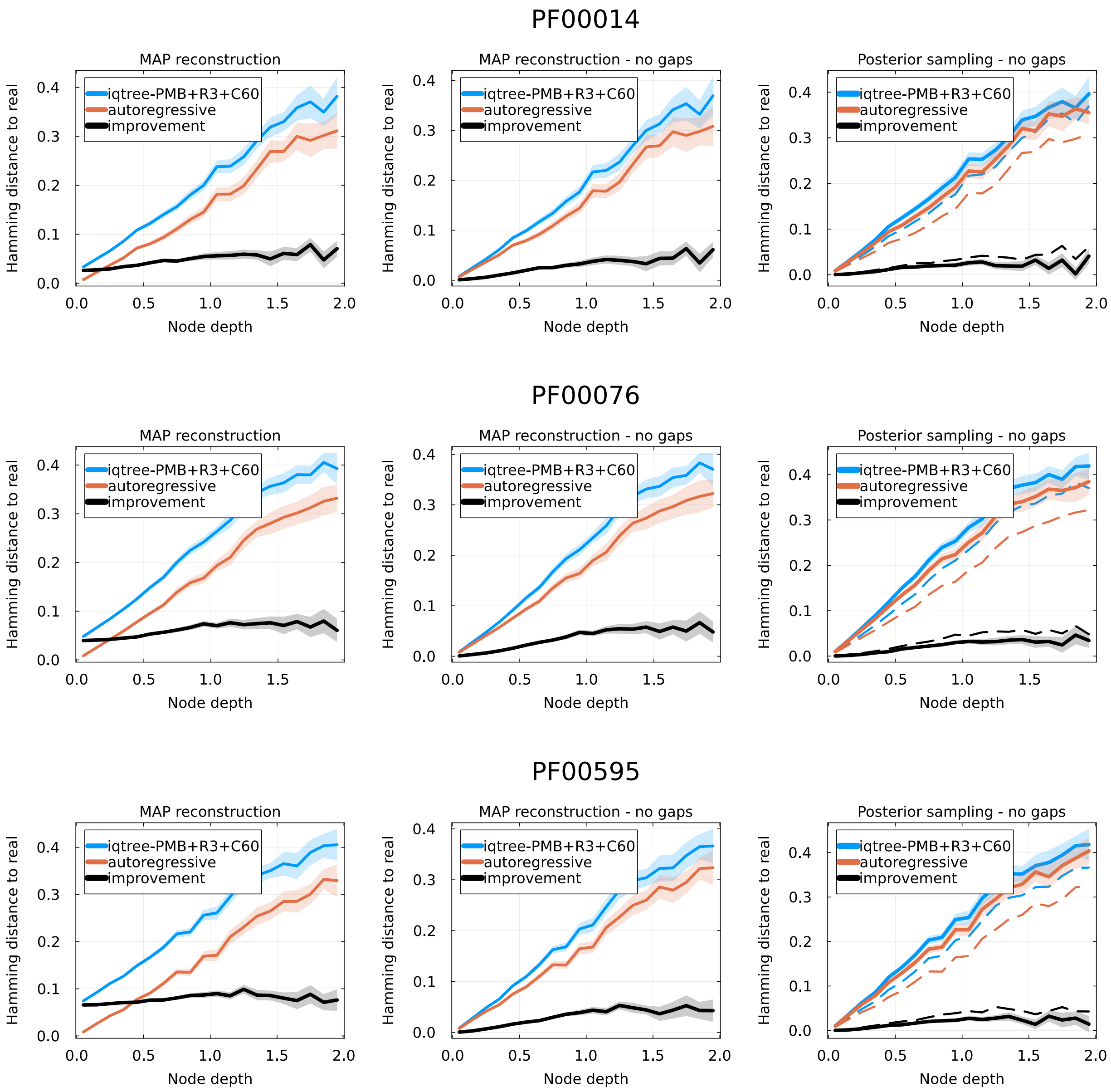

FIG. 11. Equivalent to Figure 1 of the main text using three other protein families, and using the `+C60` flag in IQ-TREE's reconstruction (profile model).

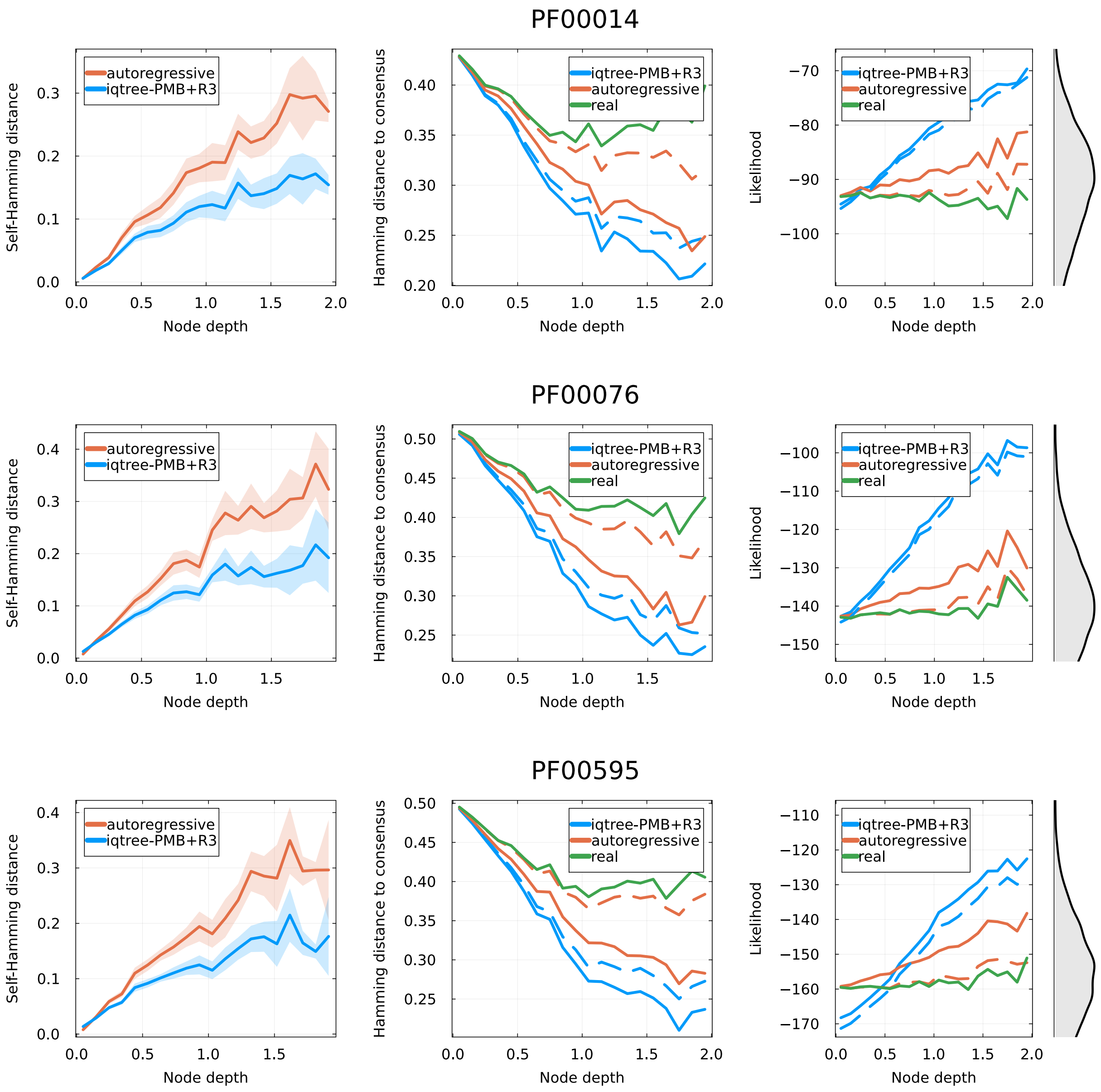

FIG. 12. Equivalent to Figure 2 of the main text using three other protein families.

Left: for posterior sampling reconstruction, average pairwise Hamming distance among sequences reconstructed for each internal node. This quantifies the diversity of possible ancestral reconstructions. **Center**: Hamming distance between reconstructed sequences and the consensus sequence of the alignment. Solid lines represent MAP reconstruction or the real internal sequences, and dashed lines posterior sampling. IQ-TREE appears more biased towards the consensus sequence. **Right**: Log-likelihood of reconstructed and real sequences in the autoregressive model, *i.e.* using the logarithm of Eq. 4. MAP methods (orange and blue solid lines) are biased towards more probable sequences. Posterior sampling autoregressive reconstruction gives sequences that are at the same likelihood level than the real ancestors. The equilibrium distribution of likelihood of sequences generated by Eq. 4 is shown on the right.

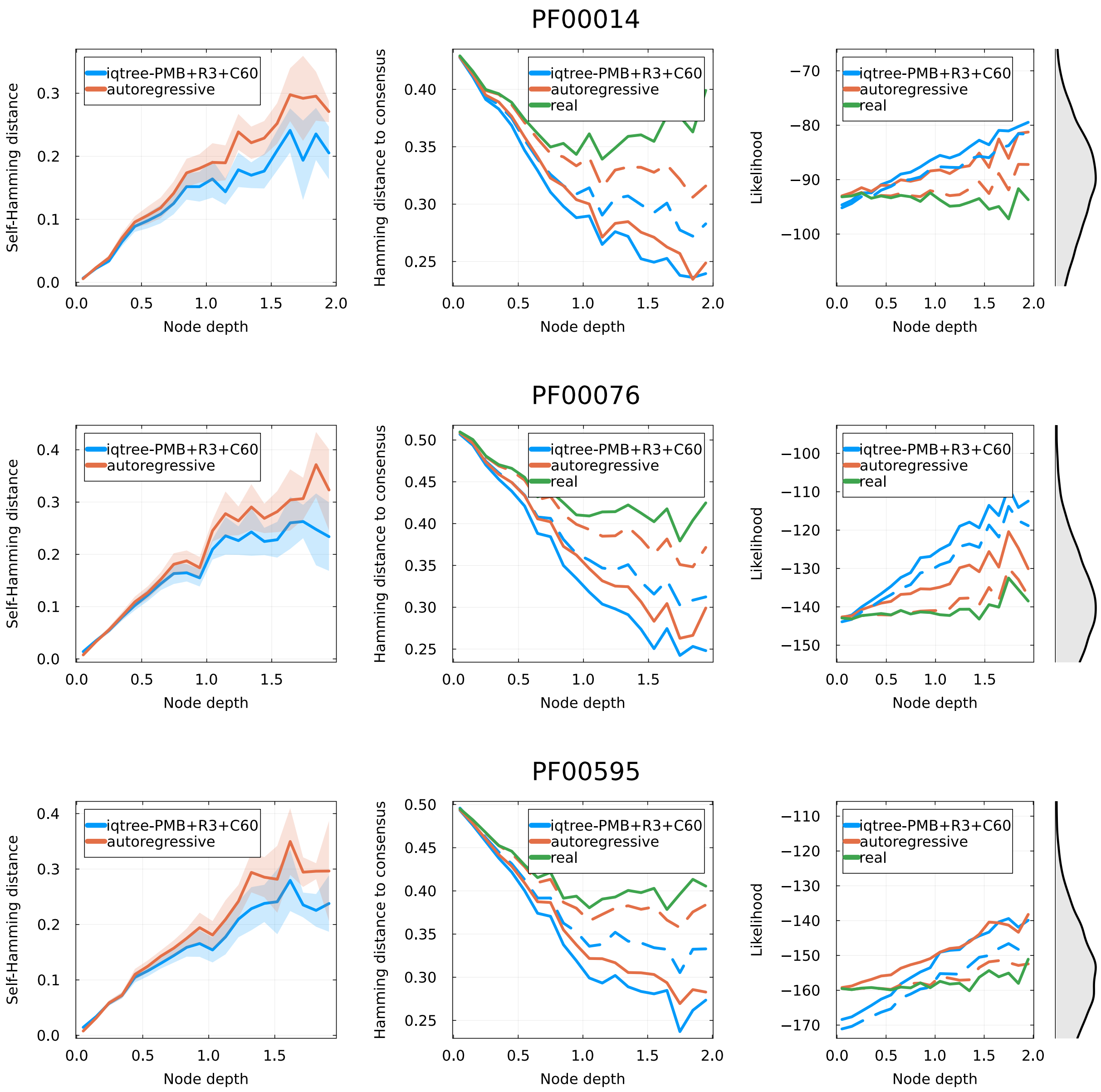

FIG. 13. Equivalent to Figure 2 of the main text using three other protein families, and using the `+C60` flag in IQ-TREE's reconstruction (profile model).

[1] Joseph Felsenstein. Evolutionary trees from DNA sequences: A maximum likelihood approach. *Journal of Molecular Evolution*, 17(6):368–376, November 1981. ISSN 0022-2844, 1432-1432. doi:10.1007/BF01734359.

[2] Bastien Boussau and Manolo Gouy. Efficient Likelihood Computations with Nonreversible Models of Evolution. *Systematic Biology*, 55(5):756–768, October 2006. ISSN 1063-5157. doi:10.1080/10635150600975218.

[3] Thomas Harvey Rowan. *Functional stability analysis of numerical algorithms.* PhD thesis, Department of Computer Science, University of Texas at Austin, Austin, TX, 1990.

[4] Steven G. Johnson. The NLopt nonlinear-optimization package. `https://github.com/stevengj/nlopt`, 2007.

[5] Sophia Alvarez, Charisse M. Nartey, Nicholas Mercado, Jose Alberto de la Paz, Tea Huseinbegovic, and Faruck Morcos. In vivo functional phenotypes from a computational epistatic model of evolution. *Proceedings of the National Academy of Sciences*, 121(6):e2308895121, February 2024. doi:10.1073/pnas.2308895121.

[6] Douglas M. Robinson, David T. Jones, Hirohisa Kishino, Nick Goldman, and Jeffrey L. Thorne. Protein Evolution with Dependence Among Codons Due to Tertiary Structure. *Molecular Biology and Evolution*, 20(10):1692–1704, October 2003. ISSN 0737-4038. doi:10.1093/molbev/msg184.

[7] Nicolas Rodrigue, Hervé Philippe, and Nicolas Lartillot. Assessing Site-Interdependent Phylogenetic Models of Sequence Evolution. *Molecular Biology and Evolution*, 23(9):1762–1775, September 2006. ISSN 0737-4038. doi:10.1093/molbev/msl041.

[8] Chris A. Nasrallah, David H. Mathews, and John P. Huelsenbeck. Quantifying the Impact of Dependent Evolution among Sites in Phylogenetic Inference. *Systematic Biology*, 60(1):60–73, January 2011. ISSN 1063-5157. doi:10.1093/sysbio/syq074.
